# Multi‐Omic Evaluation of PLK1 Inhibitor—Onvansertib—In Colorectal Cancer Spheroids

**DOI:** 10.1002/jms.5137

**Published:** 2025-04-08

**Authors:** Brian D. Fries, Emily R. Sekera, Joseph H. Holbrook, Amanda B. Hummon

**Affiliations:** ^1^ Department of Chemistry and Biochemistry The Ohio State University Columbus Ohio USA; ^2^ Ohio State Biochemistry Program The Ohio State University Columbus Ohio USA; ^3^ Comprehensive Cancer Center The Ohio State University Columbus Ohio USA

**Keywords:** cell cycle, colorectal cancer, lipidomics, mass spectrometry imaging, Plk‐1 inhibition, proteomics, spatial lipidomics, spheroids

## Abstract

Polo‐like kinase 1 (Plk1) is a serine/threonine kinase involved in regulating the cell cycle. It is activated by aurora kinase B along with the cofactors Borealin, INCE, and survivin. Plk1 is involved in the development of resistances to chemotherapeutics such as doxorubicin, Taxol, and gemcitabine. It has been shown that patients with higher levels of Plk1 have lower survival rates. Onvansertib is a competitive ATP inhibitor for Plk1 in clinical trials for the treatment of tumors and has recently entered a trial for the treatment of *KRAS* mutant colorectal cancers (CRCs). In this study, we conducted an untargeted liquid chromatography–mass spectrometry (LC–MS) proteomics study as well as an untargeted lipidomics analysis of HCT 116 spheroids treated with onvansertib over a 72‐h treatment time‐course experiment. Mass spectrometry imaging (MSI) showed that onvansertib begins to accumulate most prominently after 12 h of treatment and continues to accumulate through 72 h. Proteomic results displayed alterations to cell cycle control proteins and an increasing abundance of aurora kinase B and Borealin. The proteomics data also showed alterations to many lipid metabolism enzymes. The MSI lipidomics data indicated alterations to phosphatidylcholine lipids, with many lipids increasing in abundance over time or increasing until 12 h of onvansertib treatment and decreasing after that time point. In summary, these results suggest that onvansertib is causing cells within the spheroid to halt at a certain phase of the cell cycle in accordance with previous literature. Our findings suggest the S phase is likely interrupted, with observed alterations in cell cycle control proteins and PC lipid abundance.

## 1 Introduction

The Polo gene encodes a serine/threonine kinase first discovered in *Drosophila melanogaster* flies in 1988 by Claudio Sunkel and David Glover [1]. A homologous kinase was mapped to humans in 1994, giving rise to the class of Polo‐like kinases (Plks) [2]. There are five Plks known to exist in humans, with Plk1 being the most studied. Plk1 is activated by phosphorylation from aurora kinase A along with the cofactor Borealin [3]. Plk1 has also been shown to be activated by aurora kinase B with the cofactors Borealin, INCE, and survivin [3, 4]. Plk1 plays a very important role in regulating the cell cycle by phosphorylating CDC25 to activate the cyclin B/cdc2 complex, thus initiating cell proliferation [5]. Plk1 expression increases during the S phase of the cell cycle, followed by a peak at the G_2_‐M transition, and lastly decreases after mitosis [2]. It has been observed that patients with cancer with high levels of Plk1 expression have a lower 3‐year survival rate than those with lower expression [6]. Plk1 is involved in developed resistance of many chemotherapeutics, such as doxorubicin [7, 8, 9], taxol [10, 11], and gemcitabine [12, 13], to name a few. Understanding ways to inhibit Plk1 will help increase the efficacy of many therapeutics and has the potential to improve survival rates in patients with cancer.

Onvansertib is a competitive ATP inhibitor that is specific to Plk‐1 [14, 15]. Onvansertib has been shown to cause a dose‐dependent response in multiple different cancer cell lines [15] as well as impair cell regrowth in HT‐29 colorectal cancer (CRC) and head and neck squamous cell carcinoma (HNSCC) cells [16]. A recent Phase Ib study in patients with relapsed or refractory acute myeloid leukemia (AML) showed that onvansertib, in combination with cytarabine or decitabine, resulted in decreased levels of mutant circulating tumor DNA [17]. A Phase IB/II clinical trial is currently underway to understand onvansertib treatment in combination with FOLFIRI and bevacizumab for patients with metastatic CRC containing a KRAS oncogenic mutation [18]. Many of the initial studies with onvansertib have been completed in two‐dimensional cell cultures, which lack the complexity of an in vivo human tumor. A three‐dimensional cell culture model—such as a spheroids—provides a much better conduit to understand the pharmacokinetics of a compound.

Spheroids are a three‐dimensional cell culture technique originally developed by Robert Sutherland in the 1970s [19]. Spheroids grow in a radially symmetric manner, developing distinct cell populations as well as various chemical and nutritional gradients. The core of a spheroid consists of largely necrotic cells with an acidic pH and very low levels of oxygen [19, 20, 21, 22]. The middle layer consists of quiescent cells and the outer layer consists of proliferating cells [19]. Spheroids and other more complex cell culture systems are gaining popularity for assessing therapeutics due to the passage of the FDA Modernization Act 2.0 in 2022 [23]. Much work has been completed recently to characterize the distinct molecular differences between 2D cell cultures and 3D cell cultures [24, 25, 26, 27, 28, 29]. Prior studies have shown that there are molecular differences when monolayer cultures are treated with a therapeutic compared with their spheroid counterpart [25, 26, 29]. One of the benefits of using spheroids for therapeutic studies is the ability to determine how well a pharmaceutical compound will penetrate into a spheroid [30], as a predictor of similar behavior in a tumor. Using mass spectrometry imaging (MSI) techniques, one can simultaneously track how deep a therapeutic has penetrated into a spheroid, assess drug metabolism, and map changes to endogenous small molecules throughout the spheroid [31].

In this study, we map the spatial distribution of onvansertib in HCT 116 spheroids over 72 h using MSI. Data independent acquisition (DIA) proteomics was also conducted at each time point and the proteome and lipidome were analyzed to further understand onvansertib actions in HCT 116 CRC spheroids.

## 2 Methods

### 2.1 Chemicals

Liquid chromatography–mass spectrometry (LC–MS) grade water, acetonitrile (ACN), 0.1% formic acid in water, and 0.1% formic acid in acetonitrile were obtained from Burdick & Jackson. Dimethyl sulfoxide (DMSO) was obtained from MP Biomedicals. Sodium dodecyl sulfate (SDS), sodium orthovanadate, sodium fluoride, sodium pyrophosphate, β‐glycerophosphate, trifluoroacetic acid (TFA), 2,5‐dihydroxybenzoic acid (DHB), α‐cyano‐4‐hydroxycinnamic acid (CHCA), and triethylammonium bicarbonate (TEAB, 1 M, pH = 8.5) were obtained from Sigma‐Aldrich. Methanol was obtained from Fisher Scientific. Onvansertib (NMS‐P937) was obtained from Selleckchem. The Cell Titer Glo® 3D cell viability assay was obtained from Promega.

### 2.2 Spheroid Culture

HCT 116 cells were acquired from American Type Culture Collection (ATCC) and cultured in McCoy's 5A medium supplemented with 1% l‐Glutamine, 1% Penicillin/Streptomycin, and 10% fetal bovine serum (FBS). HCT 116 cells were grown in monolayers at 37°C in 5% CO_2_ until they reach approximately 80% confluency. Spheroids were cultured using the liquid overlay method as previously described [32, 33]. In brief, outer moats of a 96‐well plate were filled with 1X PBS. A 1.5% agarose solution made of McCoy's 5A was added to the bottom of each well and allowed to cool. HCT 116 cells in suspension were added to each well at 7000 cells per well. Seeded cells were spun at 200 g for 10 min, prior to placement in the incubator to ensure proper spheroid formation. HCT 116 spheroids were grown for four days untouched. After four days, half of the initial media volume was changed every 48 h until Day 12. Spheroids were treated with onvansertib on Day 12 and harvested at each respective time point. Harvested spheroids were washed with 1X PBS and either stored at −80°C for proteomics analysis or embedded in 20% (w/v) gelatin for MALDI‐MSI.

### 2.3 IC_50_ Determination

The 50% inhibitory concentration (IC_50_) of onvansertib was determined by using the Cell Titer Glo® 3D. Spheroids were incubated with onvansertib for 72 h in concentrations ranging from 250 to 0.120 μM. After 72 h, 100 μL of media were removed from the spheroids and 100 μL of reagent were added. The spheroids were vortexed for 5 min followed by incubation with the reagent for 25 min. The total luminescence intensity was recorded and visualized using Graph Pad Prism to calculate the IC_50_.

#### 2.3.1 MALDI‐MSI

Spheroids were sectioned at 10 μm thickness using a cryostat with a chamber and chuck head temperature of −30°C. Samples were thaw mounted onto indium tin oxide‐coated glass slides (Delta Technologies). Matrix solutions of CHCA (10 mg/mL in 50% ACN with 0.1% TFA) or DHB (10 mg/mL in 50% ACN with 0.1% TFA) were used to coat samples using an HTX Imaging M5 TM‐Sprayer. Sprayer parameters are summarized in Table S1. Samples were analyzed immediately after application of MALDI matrix.

MALDI‐MSI spectra were acquired using a Bruker timsTOF fleX with MALDI‐2. Mass spectra were acquired in positive ion mode with a mass range set to acquire between 200 and 1800 Da. The laser spot size was set to beamscan with a resulting field size of 20 × 20 μm at a frequency of 1000 Hz at 70% laser power with MALDI‐2 enabled. Spatial raster distance was set to 20 μm along the x and y axes accumulating 500 shots per pixel. External calibration was completed using Agilent ESI‐TOF calibration standard. Samples were analyzed in biological triplicate.

### 2.4 Lipid Tandem MS

HCT 116 spheroids were lysed in water using a probe sonicator. A 10‐mg/mL matrix solution of CHCA was prepared with 50% ACN and 0.1% TFA. Equal parts matrix solution and cell lysate were mixed and spotted onto a MALDI plate. Putative lipid ions discovered within the MALDI‐MSI analysis were analyzed within the timsTOF fleX mass spectrometer. The mass spectrometer was calibrated with red phosphorus. Ions were isolated in a 1.50‐*m/z* wide isolation width and fragmented with CID at varying collisional energies (ce). Lipid fragments were manually annotated for lipid identification.

### 2.5 Proteomics Sample Preparation

HCT 116 spheroids were lysed in mammalian cell lysis buffer using a probe sonicator consisting of 6% SDS, 1 mM each of sodium fluoride, sodium orthovanadate, β‐glycerophosphate, 10 mM of sodium pyrophosphate, 100 mM TEAB, and one complete EDTA‐free protease inhibitor cocktail tablet (Sigma). Approximately 400 μg of protein was aliquoted for proteomics. Samples were first reduced with 20 mM DTT for 15 min at 65°C followed by alkylation with 40 mM IAA for 30 min in the dark. Next, samples were acidified with phosphoric acid to 1.2% and diluted with 90% Methanol/100 mM TEAB in a 1:6 ratio. Proteins were spun onto the S‐Trap column at 4000 ×*g* for 1 min and washed four times with 90% methanol/100 mM TEAB. Trapped proteins were spun once at 4000 ×*g* for 1 min with no wash to remove excess methanol. S‐Trap columns were moved to a new column where Trypsin Gold/Lys‐C was added in a 1:50 trypsin:protein ratio in 50 mM TEAB and incubated in a water bath overnight at 37°C. Peptides were eluted sequentially with equal volumes of 50 mM TEAB, 0.2% formic acid in water, and 0.2% formic acid in acetonitrile. Peptides were further desalted using a Waters HLB Oasis μ elution 96‐well plate (2 mg sorbent, 30 μm particle size, 80 Å pore size) and dried down prior to storage at −80°C.

### 2.6 LC–MS

Peptides were resuspended in 0.1% formic acid (Mobile Phase A) and separated using a Waters M‐Class Acuity UHPLC using a Waters nanoEase M/Z Peptide BEH C18 column (75 μm id × 200 mm length) at 50°C with a flow rate of 0.400 μL/min. Approximately 500 ng of peptides per sample were separated using a two‐step linear gradient starting at 2% mobile phase B (0.1% formic acid in acetonitrile) to 20% B in 100 min. The percent composition of B was increased to 32% in 20 min followed by a 1‐min ramp to 95% B and held at 95% for 4 min. Next, the percent B composition was ramped down to 2% in 1 min and held at 2% for 30 min to allow column equilibration. Peptides were analyzed using DIA on a Thermo QE‐HF mass spectrometer. DIA windows were constructed within EncylopeDIA [34]. Staggered DIA wide windows were collected for each proteomics sample from 400 to 1000 *m/z* in 16.0 *m/z* wide windows with full scan resolution of 60 000 and an MS2 resolution of 30 000 along with an AGC target of 1 × 10^6^. The normalized collision energy (NCE) value was set to 27. A pooled sample was also prepared and staggered narrow DIA windows were collected in 100 *m/z* intervals from 400 to 1000 *m/z* with 4 *m/z* wide DIA windows to generate a gas phase fractionated (GPF) library [35]. All .raw LC–MS files were converted to .mzML using MSConvert with peak picking and demultiplexing [36].

### 2.7 Data Analysis

MALDI‐MSI data was analyzed using SCiLS Lab 2023c and exported to Metaspace2020 for putative identifications [37]. MALDI data was normalized using the total ion count (TIC). Segmentation of spheroids from background signal was completed using weak denoising, bisecting k‐means, and the correlation distance metric using a list of the top 30 peaks within the spheroids (Figure S15). Graphpad Prism was used to generate IC_50_ plots and determine statistical significance from exported peak areas of segmented spheroid regions. The proteomics data was analyzed by first generating a predicted library from the Human FASTA Prosit and then searching the GPF's against this library using EncyclopeDIA with the Human FASTA as the background [34, 35, 38]. The searched GPF were summarized together to generate an empirically corrected chromatogram library followed by filtering to 1% FDR with Percolator [39]. The DIA proteomics samples were searched against the empirically corrected chromatogram library with the Human FASTA as the background and filtered to 1% FDR. The resulting .elib proteomics files were summarized together with match between runs (MBR) and uploaded into Skyline [40]. Within Skyline, the proteomics results were annotated and filtered to contain a minimum of two unique peptides per protein. An MSstats output was generated, and further data analysis was conducted with MSstats and Perseus as well as base R programming [41]. Functional analysis was done using Ingenuity Pathway Analysis (IPA) [42]. Gene Set Enrichment Analysis (GSEA) was conducted using the clusterProfileR package [43].

## 3 Results

### 3.1 IC_50_ Determination and MALDI‐MSI of Onvansertib

To study onvansertib in HCT 116 spheroids, the half maximum inhibitory concentration had to be determined. This assay was completed by incubating HCT 116 spheroids on Day 12 of growth with onvansertib concentrations ranging from 250 to 0.120 μM for 72 h. The dosed spheroids were then incubated with the Cell Titer Glo 3D reagent and the total luminescent intensity was measured. The IC_50_ was determined to be 30.87 ± 12.67 μM (IC_50_ ± 95% Confidence Interval) (Figure S1). Due to the three‐dimensional nature of the spheroid, it is important to understand how well a therapeutic can penetrate the cell mass. HCT 116 spheroids were incubated with onvansertib at the calculated IC_50_ concentration and harvested at eight different time points (0 (control), 1, 4, 6, 12, 24, 48, and 72 h). The harvested spheroids were embedded in gelatin prior to being stored at minus 80°C. Spheroids were also harvested at each time point for complementary proteomics analysis.

**FIGURE 1:**
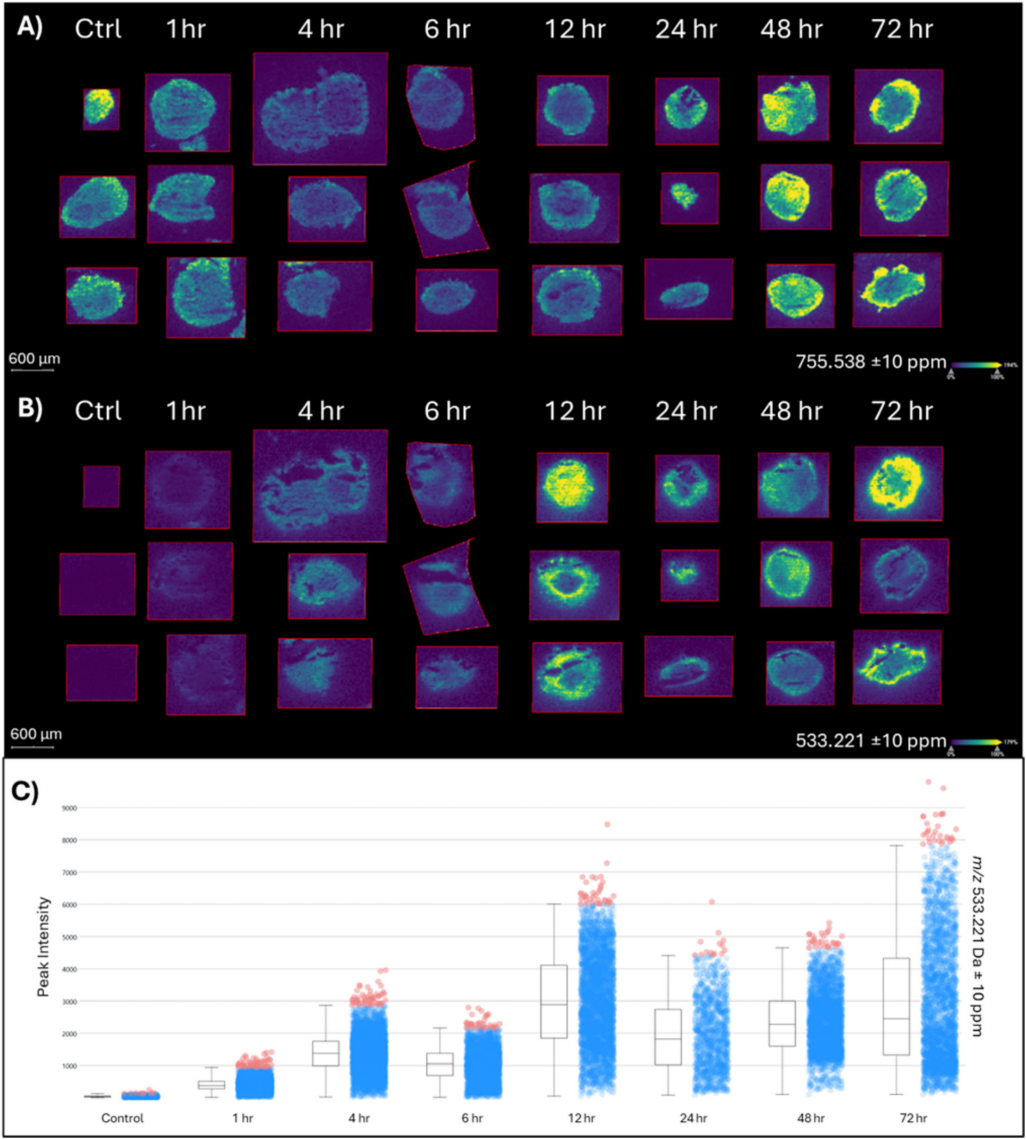
(A) Putative lipid at 755.538 Da to show the HCT 116 spheroid in the imaging data. (B) MALDI‐MSI of onvansertib (*m/z* = 533.221 Da) over the experimental time series of 0 to 72 h. (C) Box and whisker cloud plot for onvansertib.

Embedded spheroids were sectioned and placed onto the conductive side of an indium tin oxide slide. CHCA matrix in 50% ACN was sprayed onto the slides using an HTX Sprayer. Spheroids were analyzed with a Bruker timsTOF fleX with and without MALDI‐2 postionization. Each condition was analyzed in biological triplicate. The collected MSI spectra were analyzed with SCiLS lab and the spatial distribution of a putative lipid (to indicate the shape of the spheroid, Figure 1A) and onvansertib ([M + H^+^] = 533.221 Da) were visualized. As shown in Figure 1B,C, onvansertib begins to accumulate in the outer regions of the spheroid after only 1 h. Onvansertib begins to more strongly penetrate the spheroid at 12 h after incubation, where it then continues to accumulate throughout the entire spheroid. While a slight decrease in signal is observed, after 12 h, onvansertib accumulation continues through the 72‐h timepoint.

### 3.2 Proteomics

Proteins harvested from spheroids at each time point were digested into peptides using S‐Trap columns. Peptides were analyzed on a QE‐HF mass spectrometer using staggered DIA with 16 *m/z* wide windows. A pooled sample was also prepared, and narrow window DIA spectra were collected over 100 *m/z* ranges from 400 to 1000 *m/z* with 4 *m/z* wide windows. The narrow window DIA was searched against an in silico Prosit‐made spectral library and compiled together to have an empirically corrected gas phase fractionated library (EMP‐GPF) [35]. The DIA proteomics samples were searched with the EMP‐GPF library. A total of 72 249 peptides were identified and filtered to a 1% FDR [35]. After further filtering in Skyline and MSstats to require each protein contain two or more peptides and those peptides to be unique, 5751 proteins were quantified. Figure S2 shows consistent normalized and log_2_ transformed MS1 TIC levels. Hierarchical clustering shows that the 72‐h time points cluster entirely together (Figure S3). The other time points did not cluster distinctly from each other.

**FIGURE 2:**
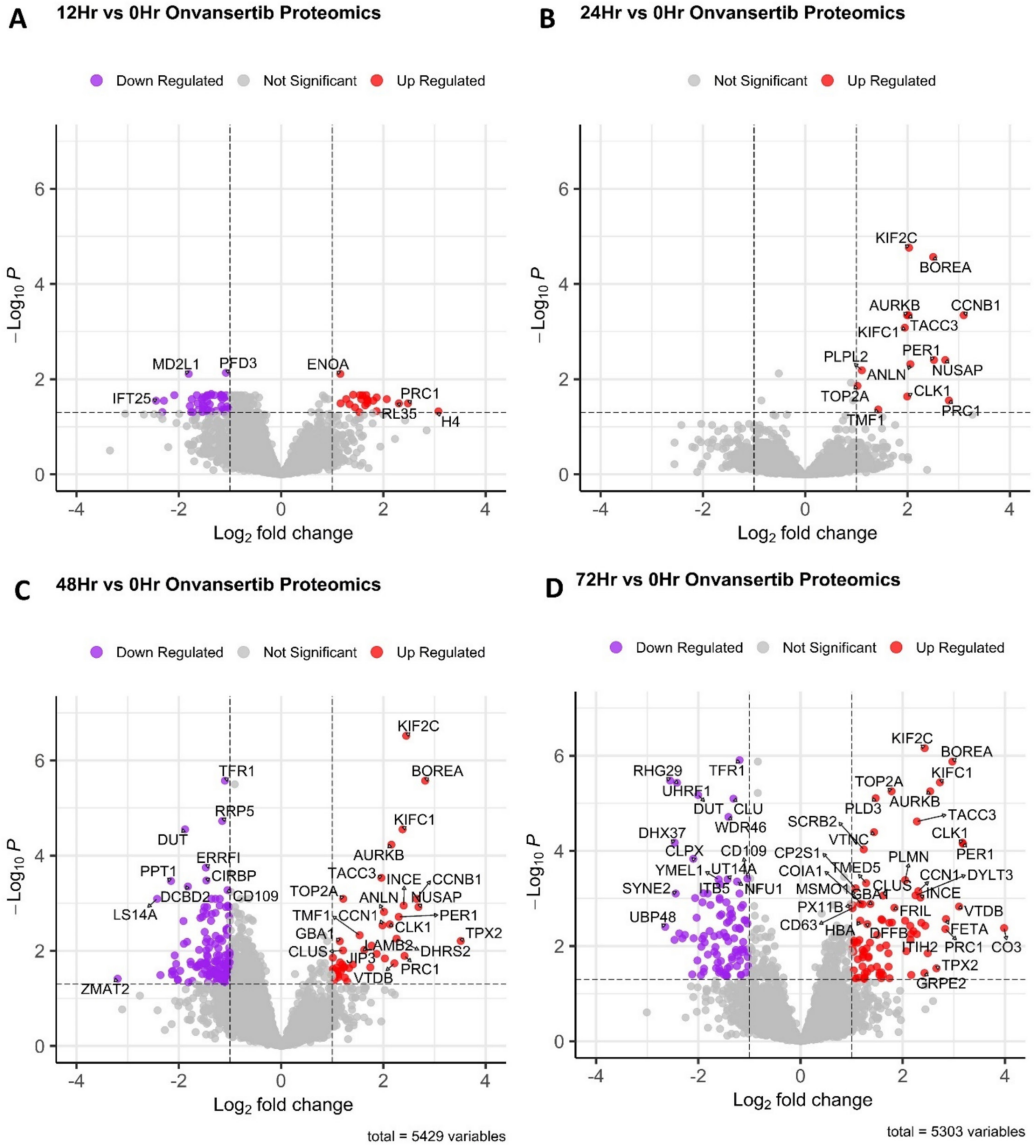
Differentially expressed proteins found within the (A) 12‐h onvansertib exposure, (B) 24‐h onvansertib exposure, (C) 48‐h onvansertib exposure, and (D) 72‐h onvansertib exposure.

**FIGURE 3:**
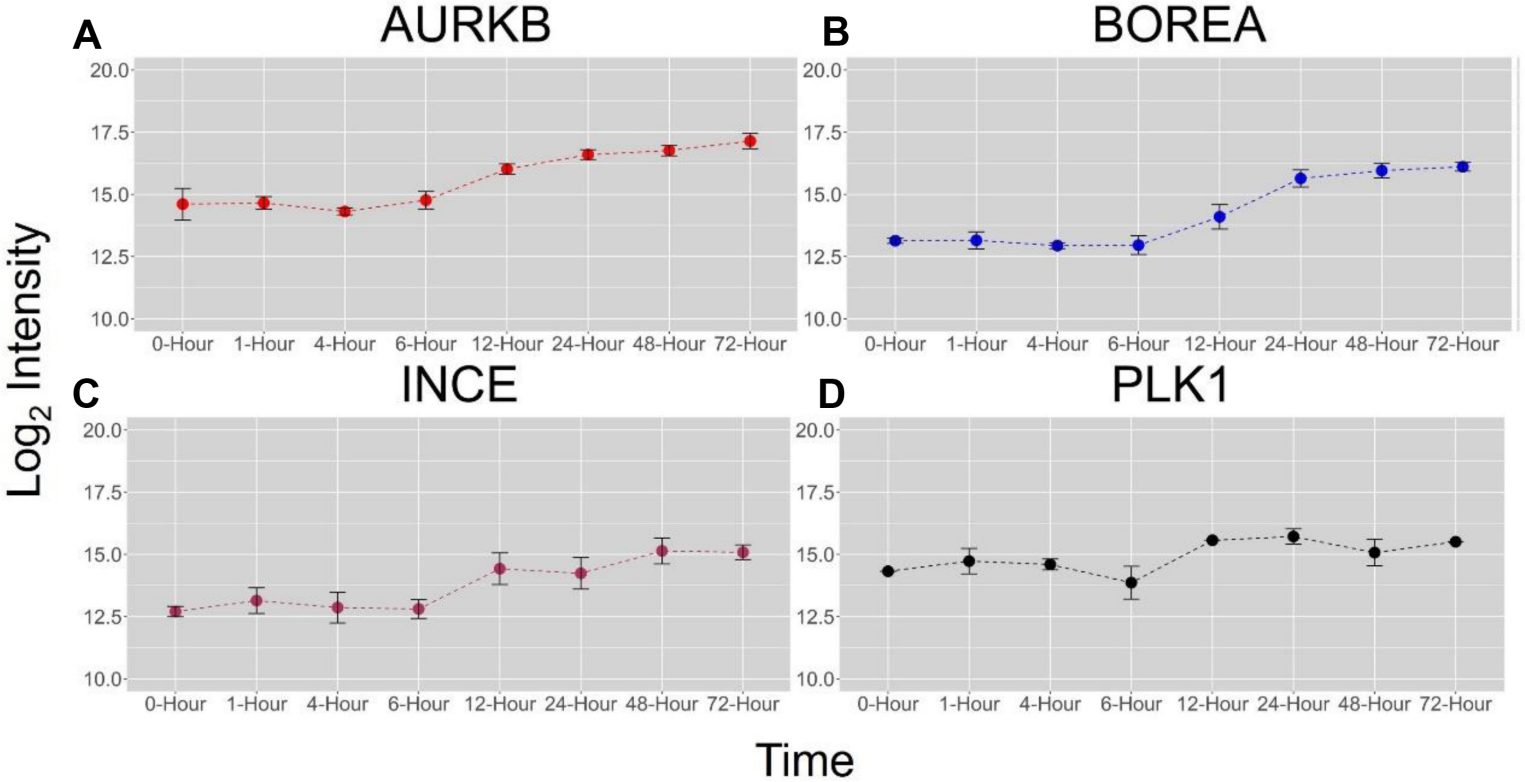
Time course changes of select proteins represented as their log_2_ intensities against the respective time point.

Next, each time point was compared against the 0‐h control time point to determine which proteins were differentially expressed (log_2_FC ≥ 1 or ≤ − 1 and *p*‐value < 0.05). The Benjamini–Hochberg hypothesis testing correction approach was applied to the *p* values to account for false positives [44]. It was observed that all the early time points (1, 4, and 6 h) had no differentially expressed proteins (Figure S4A–C). The late time points (12, 24, 48, and 72 h) were observed to have a changing number of differentially expressed proteins over time as the onvansertib was consumed by the cells (Figure 2A–D). Comparison of the differentially expressed proteins among all four time points showed that only eight proteins were similar among all three conditions (Figure S5). Aurora kinase B (AURKB) was found to be upregulated in all four time points (Figure 3A) as well as other proteins involved in the cell cycle process, such as CCNB1, KIFC1, and PRC1 (Supplemental Table 2). No proteins were shared among the 12‐, 24‐, and 72‐h time points (Figure S5). One of the cofactors of aurora kinase B, Borealin (BOREA), was found to be up‐regulated in the 24‐, 48‐, and 72‐h time points with an increasing intensity over time (Figure 3B). The INCE cofactor was detected in all samples, and only differentially expressed in the 12‐, 48‐, and 72‐h time points (Figure 3C). In the 24‐h time point, INCE had a log_2_FC = 1.500, but was not statistically significant. The last cofactor, survivin, was not identified. Aurora kinase A was also not identified within the proteomics data set. Plk1 (Figure 3D) was detected in this data and was elevated in the 12‐, 24‐, and 72‐h time points (log_2_FC > 1); however, Plk1 was not elevated in the 48‐h time point (log_2_FC = 0.957). Plk1 levels were not statistically significant (*p* > 0.05) in all time points compared to the 0‐h time point.

**FIGURE 4:**
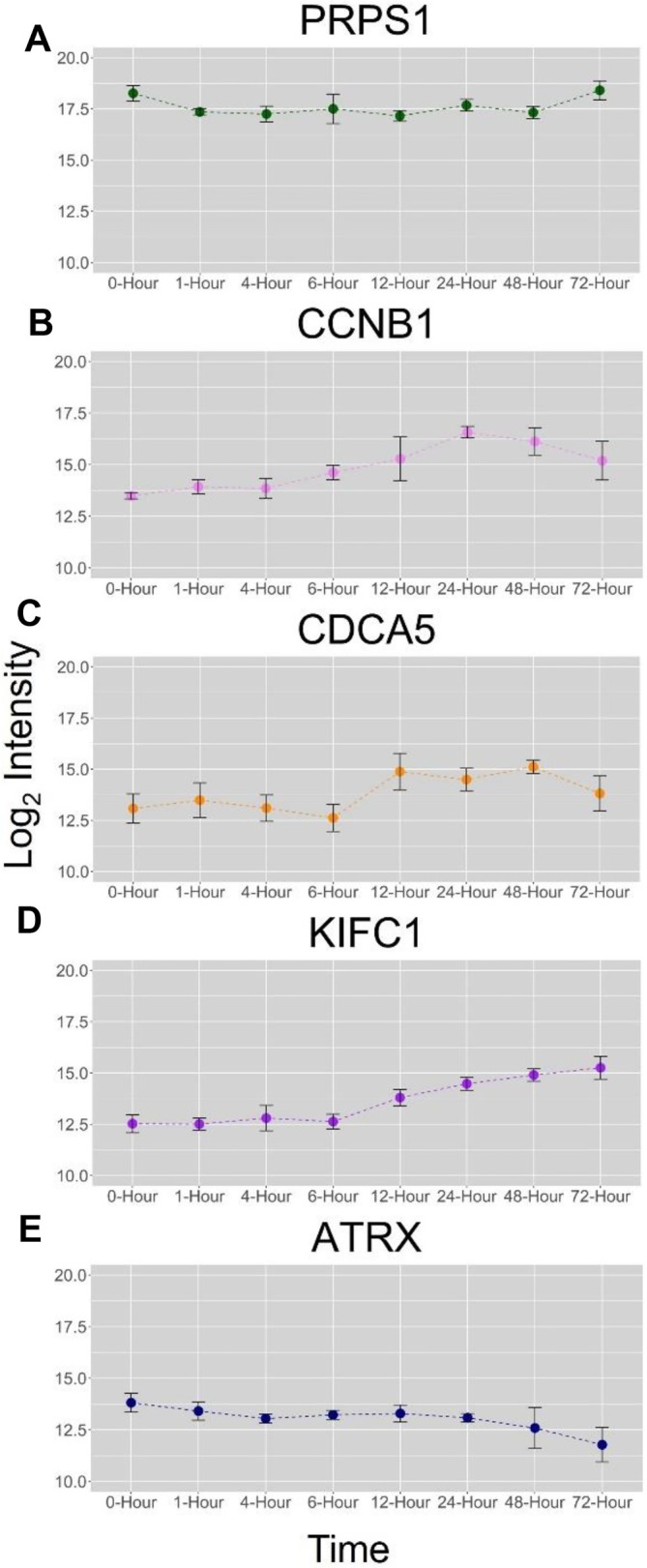
Time course changes of select proteins represented as their log_2_ intensities against the respective time point.

The 12‐h time point was observed to have 21 up‐ and 46 down‐regulated proteins, respectively, with 41 of these proteins found to be unique to this time point (Figures 2A and S5). A pathway analysis conducted in IPA shows that the 12‐h time point only had predicted activation of one pathway, the Intra Golgi and retrograde Golgi‐ER traffic (Figure S10). GSEA analysis shows activation and suppression of many different gene sets (Figure S6). Cellular response to nitrogen, organonitrogen, and oxygen containing compounds were suppressed (Figure S6). This suppression is exemplified by the down regulation of PRPS1 (Figure 4A), an enzyme that catalyzes the synthesis of phosphoribosyl pyrophosphate, which is required for the synthesis of nucleotides. GSEA analysis also showed activation of ribosome and structural constituent of ribosome gene sets (Figure S6). This enrichment can be observed in the proteomics data, where the 12‐h time point had unique upregulation of three different ribosomal subunit proteins, RL35, RS6, and RS7 (Supplementary Table S1).

The 24‐h time point interestingly contained only upregulated proteins when compared against the 0 h control (Figure 2B). Only one protein was unique to this time point, PLPL2, an enzyme involved in hydrolyzing triglycerides from lipid droplets (Figure 2). IPA analysis predicted no activated pathways among the differentially expressed proteins, and GSEA analysis only showed nine altered gene sets (Figure S7). Regulation of cell cycle process and regulation of cell cycle gene sets were activated, and catalytic activity gene sets were suppressed (Figure S7). The most upregulated protein in the 24‐h time point was CCNB1 (Figure 4B), which is involved in control of the cell cycle during the G_2_/M transition.

The 48‐h time point contained many more altered proteins with 43 up‐ and 144 down‐regulated proteins (Figure 2C). GSEA analysis shows activation of many cell cycle processes, such as positive regulation of cytokinesis, mitotic spindle elongation, and regulation of mitotic cytokinesis gene sets (Figure S8). However, there was suppression of gene sets related to nucleobase‐containing small molecule metabolic process, organophosphate biosynthetic process, nucleotide metabolic process, and nucleotide biosynthetic process (Figure S8). IPA analysis shows predicted activation of mitotic metaphase and anaphase pathways as well as mitotic prometaphase (Figure S10). There was predicted deactivation of rRNA processing in the nucleolus and cytosol (Figure S10). Specific proteins involved in the cell cycle were upregulated and unique to this timepoint, such as CDCA5 (Figure 4C).

Many differentially expressed proteins were found in the 72‐h time point with 85 up‐ and 98 down‐regulated proteins (Figure 2). Many GO terms for cell division processes were enriched after GSEA analysis, such as organelle fission, cell division, chromosome segregation, and nuclear division gene sets (Figure S9). This enrichment may be the result of up regulation of KIF11, KIF23, KIF2C, and KIFC1 (Figure 4D), all proteins required for proper cell division. The three largest containing gene sets from this time point were all suppressed terms: nucleic acid binding, nuclear lumen, and cellular nitrogen compound metabolic process (Figure S9). This response is apparent in the specific downregulation of proteins involved in DNA and RNA synthesis, such as ATRX (Figure 4E), and PRI2. It was observed that 72‐h time points had a lower level of predicted activation of mitotic metaphase and anaphase compared to the 48‐h time points (Figure S10).

**FIGURE 5:**
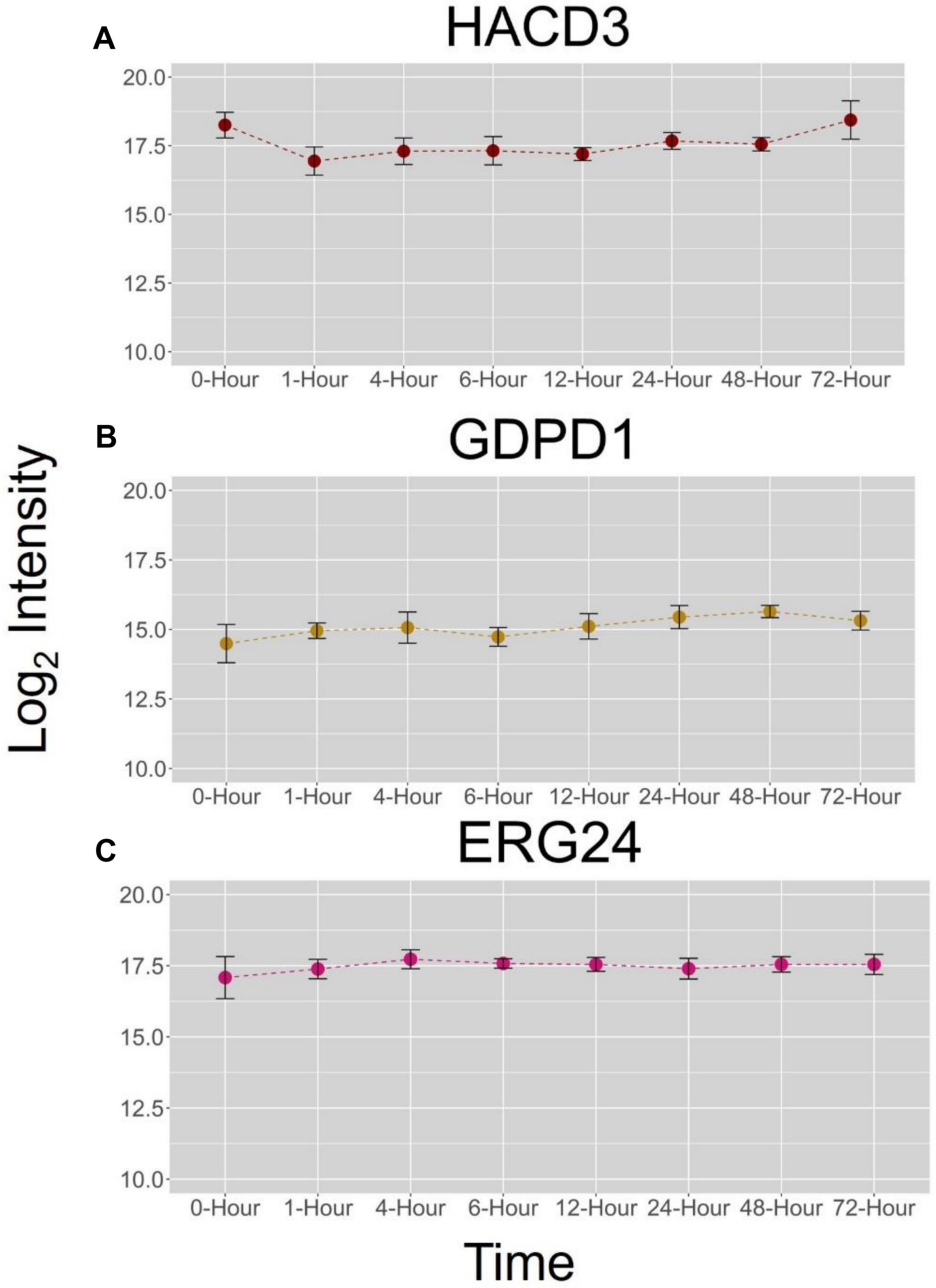
Time course changes of select proteins represented as their log_2_ intensities against the respective time point.

Changes to many enzymes involved in lipid metabolism were also observed in each of the four late time points. In the 12‐h time point, the enzyme HACD3—which catalyzes the third reaction of the very long chain fatty acid elongation cycle—was observed to be downregulated (Figure 5A), suggesting that there is alteration to lipid metabolism with onvansertib exposure (Supplementary Table S1). Other lipid metabolism enzymes were observed in the 48‐ and 72‐h time points, such as upregulation of GAB1, which cleaves glucose from glucosylceramides to releases free ceramides into the cell and downregulation of AGAL, an enzyme that hydrolyzes glycosphingolipids. The 48‐h time point was observed to have upregulation of lysophospholipase D (GDPD1) (Figure 5B). The 72‐h time point had up regulation of phosphatidylserine synthase 1 (PTSS1) and ERG24 (Figure 5C), an enzyme involved in cholesterol synthesis. The alteration to many lipid synthesis enzymes prompted us to inspect the MSI data closer to determine if various lipid species could be visualized and alterations to their expression observed over time.

### 3.3 Spatial Lipidomics

Spheroids from each time point were embedded in gelatin and sectioned onto conductive ITO slides. The sectioned spheroids were imaged using a Bruker timsTOF fleX with and without MALDI‐2 in positive ion mode using either CHCA or DHB matrix. For each time point, three different spheroids were imaged for a total of 24 total spheroids imaged across the entire experiment. The MSI data were processed using SCiLS to determine various peaks of interest. Further annotation of peaks of interest were validated by conducting tandem MS on HCT 116 spheroid lysate and each MS/MS spectrum was manually annotated to discern lipid head group identity, total number of carbons, and degree of unsaturation.

**FIGURE 6:**
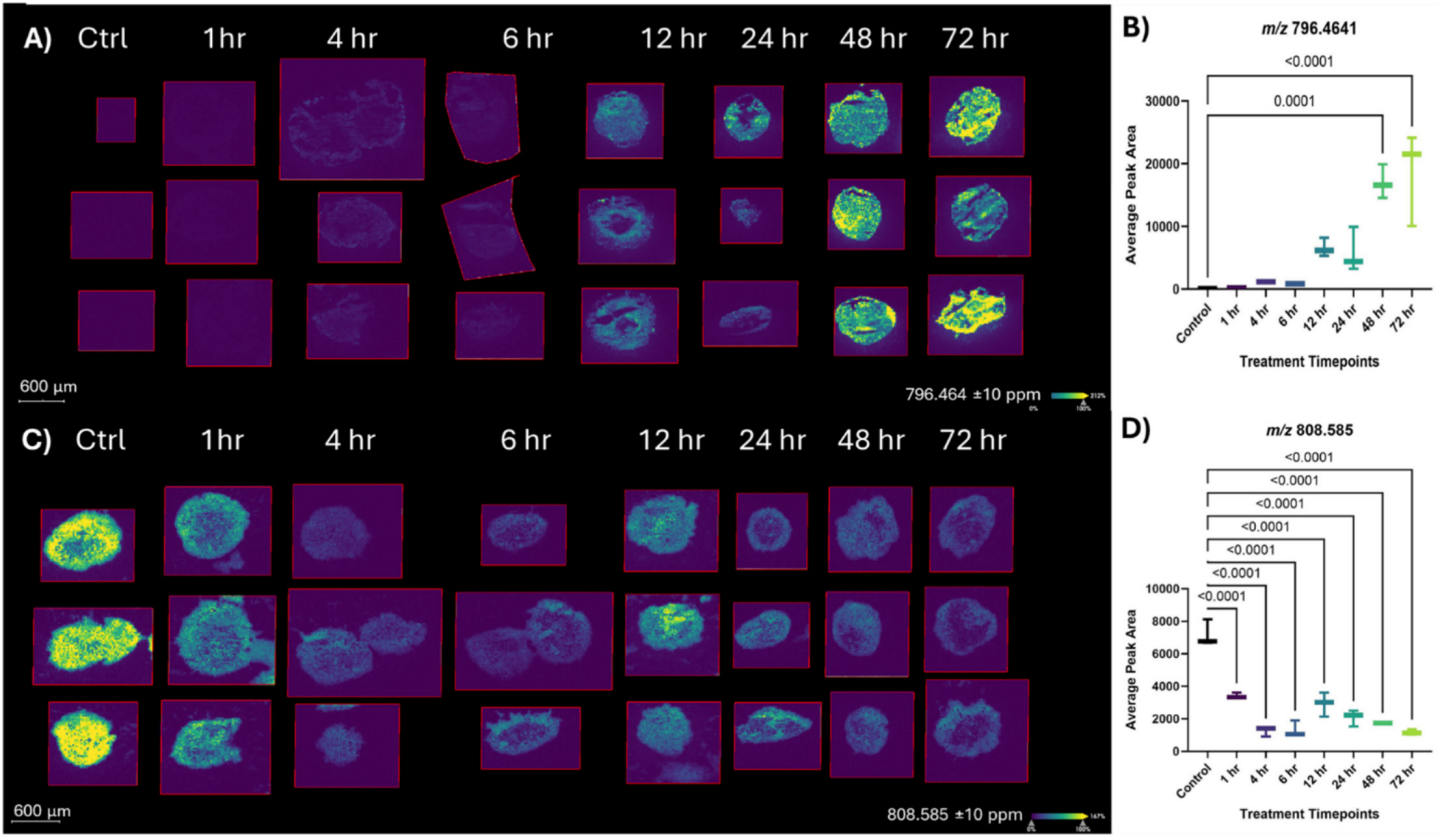
Changing abundance of lipids after onvansertib exposure. (A) Increasing intensity of *m/z* 796.464 Da identified as the [M + K]^+^ of PC(34:2) using MALDI‐1 with average peak areas plotted and statistical analysis shown for comparison against controls (B). (C) Decreasing intensity of *m/z* 808.585 Da identified as the [M + K]^+^ of PC(36:10) LIPID using MALDI‐2 with average peak areas plotted and statistical analysis completed against controls (D).

Many lipid peaks were observed with MALDI‐1 in the presented data. Some lipids, such as *m/z* 796.464, were observed to not be present or have very low intensity in the 0‐h time point and then gradually increase over time with onvansertib treatment (Figure 6A). The average peak area for the 48‐ and 72‐h timepoints were found to be statistically significant at 0.0001 and < 0.0001, respectively (Figure 6B). Based on characteristic fragments, accurate mass, and prior observations, the *m/z* 796.464 in Figure 6A is identified as the [M + K]^+^ of PC(34:2) (Figure S13A) [45]. Other lipid species were observed to have relatively high intensity from 0‐ to 12‐h followed by rapid decrease in intensity at 24‐ through 72‐h (Figure S11A). The *m/z* 798.542 shown in Figure S11A was annotated to be the [M + K]^+^ of PC(34:1) based on tandem MS as well as accurate mass and prior observations (Figure S13B) [46]. Lastly, there were certain lipid species that were low in intensity throughout the spheroid from 0‐ to 6‐h followed by a peak in intensity at 12‐h and then a sharp decrease in intensity from 24‐h on (Figure S11B). Tandem MS confirmed the *m/z* 770.513 observed in Figure S11C to be the [M + K]^+^ of PC(32:1) along with prior confirmation of this lipid by MALDI (Figure S13C) [47]. In both of these lipids, statistical significance was only observed between the control and 12‐h timepoint. While a putative increase was observed at 72 h, it was not found to be statistically significant.

MALDI‐2 is a relatively new ionization technique that can be utilized to observe ions that do not ionize readily with ablation from a single MALDI source. MALDI‐2 generates ions with a second UV laser pulse (~266 nm) passing through the ion plume generated from the initial MALDI ablation at a 90° angle [48]. This second laser pulse allows ionization of certain analytes that are not typically ionizable by the initial MALDI ionization process. MALDI‐2 has been shown to ionize various sterols with great efficiency, such as cholesterol [49]. Cholesterol was a lipid of interest for this data set with the differential expression of various enzymes involved in cholesterol synthesis and trafficking (Supplemental Table S2). Analysis of *m/z* 369.351 Da, identified as the [M + H^+^‐H_2_O]^+^ of cholesterol based on prior lipidomic acquisitions, showed a gradual increase in intensity up until 4 h after onvansertib exposure, followed by a decrease in intensity over time after 4 h (Figure S12) [49, 50, 51]. Statistical analysis of the peak areas found the 4‐h timepoint to be the only timepoint significantly altered in comparison to the control dataset. In comparison, the later time points of 12‐, 48‐, and 72‐h were all found to be lower than the 4‐h time point with statistical significance. Examination of *m/z* 808.585—identified as the [M + K^+^]^+^ of PC(36:10) using MALDI‐2 ionization (Figures 6C and S14)—started at a very high intensity, but as the time exposed to onvansertib increased, the intensity of the lipid decreased over time. Statistical significance was observed between the control dataset and all other time points (Figure 6D).

## 4 Discussion

We have conducted a time course MALDI‐MSI and untargeted proteomics study of HCT 116 spheroids exposed to the Plk1 inhibitor onvansertib. Plk1 is a protein kinase that is involved in controlling the cell cycle. It has been reported that patients with cancer with elevated levels of Plk1 have poor survival rates [6]. Plk1 expression has also been linked to a cancer's developed resistance to many therapeutics. Onvansertib is a recently developed competitive inhibitor of ATP that is specific for Plk1 [14, 15]. Though onvansertib has shown success in clinical trials, it has yet to be determined what distinct molecular differences are caused by Plk1 inhibition by onvansertib to complete the understanding of this therapeutics’ role in cancer treatment. Using DIA proteomics, more than 5000 proteins were identified and quantified. Changes to many cell cycle control proteins were observed as well as changes to proteins involved in lipid metabolism. Using MALDI‐MSI, various lipids were then analyzed, and their changing intensity over time was observed.

Aurora kinases are serine/threonine kinases with high levels of sequence similarity among the three members and play distinct roles within the cell [52]. The main role of AURKB is to be a component of the chromosomal passenger complex (CPC), the master controller of cell division. The CPC regulates chromosome structure, spindle assembly checkpoints, as well as kinetochore‐microtubule attachments and is formed by AURKB, BOREA, INCE, and survivin coming together [53]. AURKB has been shown to activate Plk1 along with the other CPC proteins, with AURKB phosphorylation of Plk1 maintaining Plk1 activity at the kinetochore to ensure accurate chromosome segregation [3, 4]. It was observed in the following data that AURKB, BOREA, and INCE had increasing log_2_FCs and increased statistical significance over time with onvansertib exposure (Figure 2 and Supplemental Table S2). As Plk1 is inhibited by onvansertib, it is possible that the cell is attempting to synthesize more members of the CPC over time to alleviate the inhibition and continue the process of cell division. This may also be shown by activated gene sets for ribosomes at 12 h, followed by activation of protein kinase binding after 24 h, and lastly enrichment of the CPC gene sets after 48 h. As onvansertib begins clearing out of the spheroid after 72 h, it is possible that normal cell division may be able to continue with activation of the cell division and mitotic cell process gene sets (Figure S9).

Many gene sets related to nitrogen compound synthesis were shared among the 12‐, 24‐, 48‐, and 72‐h time points. The first began at 12 h of onvansertib exposure with suppression of the cellular response to organonitrogen compound and cellular response to nitrogen compound gene sets (Figure S6). Then, after 24 h, the catalytic activity gene set was suppressed (Figure S7). The most profound change occurred after 48 h, with suppression of nucleobase‐containing small molecule metabolic process, organophosphate biosynthetic process, nucleotide metabolic process, nucleotide biosynthetic process, and lastly nucleoside phosphate biosynthetic process (Figure S8). Lastly, 72 h after onvansertib exposure, suppression of cellular nitrogen compound metabolic process was observed. However, other terms related to DNA/RNA were suppressed, such as ncRNA metabolic process, RNA binding, nucleic acid binding, and rRNA processing (Figure S9 and Figure S10). It has been shown that Plk1 plays a role in DNA synthesis during the S phase of the cell cycle by phosphorylating Orc2 at Serine 188 [12, 54]. With Plk1 being inhibited by onvansertib, DNA synthesis is stalled. It has also been shown that cellular metabolism plays a very important role in regulating the cell cycle [55]. The proteomics data along with the GSEA analysis show that nucleotide metabolism is also impaired, suggesting that genesis of various RNAs and nucleotide triphosphates is also hindered. These results show that Plk1 inhibition has over encompassing effects on cell metabolism that can alter the genesis of other cellular components.

Lipids play a vital role in cancer biology and are constantly changing as cancerous cells move through their biological processes [56]. It has been proposed that lipids may help shape the cellular cycle [57, 58, 59]. For example, it has been shown that PC lipids will typically increase in the S phase of the cell cycle and PI lipids will decrease in this phase while the cell enlarges [58]. It has also been shown that PE, PS, and PI lipids will attain their highest abundance during the G_2_/M transition in the cellular cycle [58]. Using MALDI‐MSI, shotgun lipidomics data was collected for each spheroid at each experimental time point and a handful of various lipid ions were discovered and annotated with tandem MS. A majority of the lipids identified in this experiment were PC lipids, more than likely due to PC lipids preference for ionization in positive mode MS. [60] With the trends observed for the PC lipids (Figure 4 and Figure S11A,B), onvansertib may be halting the cells' ability to exit the S phase of the cell cycle, causing a buildup of cells that are enlarging and trying to enter the G_2_ phase. The stark differences observed for PC(34:2) and PC(34:1) (Figure 6 and Figure S11A, respectively) over time are intriguing. The addition of a double bond to this lipid implies further remodeling of lipid metabolism and possibly membrane structure. More experiments will need to be conducted to understand onvansertib's role in lipid double bond changes.

## 5 Conclusion

We have conducted both an untargeted proteomics and lipidomics experiment with the Plk1 inhibitor onvansertib. A multitude of alterations were observed at both the proteomic and lipidomic levels over time. The majority of proteins altered were involved in controlling various points of the cell cycle as well lipid metabolism enzymes, with the noteworthy increase over time of the AURKB and BOREA, two cofactors required for Plk1 activation. Alterations to PC lipids were observed, with the identified PC lipids increasing over time. As many cell cycle control proteins were elevated and PC lipids, known to be involved in S phase of the cell cycle, were elevated, it is suggested that onvansertib is halting the cells in the S phase of cell growth and causing cells to continually grow and expand but no longer enter the G_2_ phase of growth.

## Author Contributions

B.D.F. and E.R.S. designed the experiments and collected the proteomics and MSI data. B.D.F. analyzed the proteomics data. B.D.F., J.H.H., and E.R.S. analyzed the MSI data. B.D.F., E.R.S., and A.B.H. wrote the manuscript.

## Conflicts of Interest

The authors declare no conflicts of interest.

## Supporting information


**Figure S1** IC_50_ curve of HCT 116 spheroids incubated with onvansertib for 72 h.
**Table S1** Spray parameters for MALDI‐MSI experiments with either DHB or CHCA.
**Figure S2** Normalized MS1 TIC for log_2_ transformed intensity values for LC–MS proteomics.
**Figure S3** Hierarchical clustering of onvansertib proteomics data showing that 72 h with onvansertib exposure cluster entirely together.
**Figure S4** Volcano plots of (A) 1 h, (B) 4 h, and (C) 6 h onvansertib exposure, showing only one differentially expressed protein among these entire time sets.
**Figure S5** Venn Diagram of similar proteins between the 12‐, 24‐, 48‐, and 72‐h samples.
**Figure S6** Gene set enrichment analysis of the differentially expressed proteins after 12 h of onvansertib exposure.
**Figure S7** Gene set enrichment analysis of the differentially expressed proteins after 24 h of onvansertib exposure.
**Figure S8** Gene set enrichment analysis of the differentially expressed proteins after 48 h of onvansertib exposure.
**Figure S9** Gene set enrichment analysis of the differentially expressed proteins after 72 h of onvansertib exposure.
**Figure S10** Ingenuity pathway analysis of statistically significant proteins among the 12‐, 24‐, 48, and 72‐h time points.
**Figure S11** Two different lipids that are not highly present in the early time points but gradually increase over time with onvansertib exposure. (A) *m/z* 798.542 identified to be the [M + K^+^]^+^ of PC(34:1). (B) *m/z* 770.513 identified to be the [M + K^+^]^+^ of PC(32:1).
**Figure S12** *m/z* 369.351, identified as the [M + H^+^‐H_2_O]^+^ of cholesterol, as observed with MALDI‐2 ionization.
**Figure S13** MALDI‐MSMS of lipids found in the mass spectrometry images for validation of lipid identity. The above tandem MS spectra originate from the following precursors: (A) *m/z* 796.464, (B) *m/z* 798.542, and (C) *m/z* 770.513.
**Figure S14** MALDI‐2 MSMS of lipids found in the mass spectrometry images for validation of lipid identity. The above tandem MS spectra originate from the precursor *m/z* 808.585.

**Data S2** Supplemental Table 2

**Data S3** Supplemental Table 2

**Data S4** Supplemental Table 2

## Data Availability

Raw LC–MS proteomics files have been uploaded to the Pride repository under PXD054453.
