# Supplementary material for: Multi‐Omic Evaluation of PLK1 Inhibitor—Onvansertib—In Colorectal Cancer Spheroids
Source: J Mass Spectrom. 2025 Apr 8;60(5):e5137. doi: 10.1002/jms.5137 (PMC11976698; doi:10.1002/jms.5137)
Supplement: Supplementary file 1 — Figure S1 IC50 curve of HCT 116 spheroids incubated with onvansertib for 72 h. Table S1 Spray parameters for MALDI‐MSI experiments with either DHB or CHCA. Figure S2 Normalized MS1 TIC for log2 transformed intensity values for LC–MS proteomics. Figure S3 Hierarchical clustering of onvansertib proteomics data showing that 72 h with onvansertib exposure cluster entirely together. Figure S4 Volcano plots of (A) 1 h, (B) 4 h, and (C) 6 h onvansertib exposure, showing only one differentially expressed protein among these entire time sets. Figure S5 Venn Diagram of similar proteins between the 12‐, 24‐, 48‐, and 72‐h samples. Figure S6 Gene set enrichment analysis of the differentially expressed proteins after 12 h of onvansertib exposure. Figure S7 Gene set enrichment analysis of the differentially expressed proteins after 24 h of onvansertib exposure. Figure S8 Gene set enrichment analysis of the differentially expressed proteins after 48 h of onvansertib exposure. Figure S9 Gene set enrichment analysis of the differentially expressed proteins after 72 h of onvansertib exposure. Figure S10 Ingenuity pathway analysis of statistically significant proteins among the 12‐, 24‐, 48, and 72‐h time points. Figure S11 Two different lipids that are not highly present in the early time points but gradually increase over time with onvansertib exposure. (A) m/z 798.542 identified to be the [M + K+]+ of PC(34:1). (B) m/z 770.513 identified to be the [M + K+]+ of PC(32:1). Figure S12 m/z 369.351, identified as the [M + H+‐H2O]+ of cholesterol, as observed with MALDI‐2 ionization. Figure S13 MALDI‐MSMS of lipids found in the mass spectrometry images for validation of lipid identity. The above tandem MS spectra originate from the following precursors: (A) m/z 796.464, (B) m/z 798.542, and (C) m/z 770.513. Figure S14 MALDI‐2 MSMS of lipids found in the mass spectrometry images for validation of lipid identity. The above tandem MS spectra originate from the precursor m/z 808.585. [file JMS-60-e5137-s004.docx]

**Multi-omic Evaluation of PLK1 Inhibitor—Onvansertib—in Colorectal Cancer Spheroids**

Brian D. Fries^1^, Emily R. Sekera^1^, Joseph H. Holbrook^2^, and Amanda B. Hummon^1,3^

1. Department of Chemistry and Biochemistry, The Ohio State University, Columbus, OH, United States of America 43210
2. Ohio State Biochemistry Program, The Ohio State University, Columbus, OH, United States of America 43210
3. Comprehensive Cancer Center, The Ohio State University, Columbus, OH, United States of America 43210

**Keywords: Plk-1 inhibition, Colorectal Cancer, Spheroids, Proteomics, Lipidomics, Mass Spectrometry Imaging, Cell Cycle, Spatial Lipidomics**

**Figure S1** IC_50_ curve of HCT 116 spheroids incubated with onvansertib for 72 hours…………...1

**Table S1** Spray parameters for MALDI MSI experiments with either DHB or CHCA……………..2

**Figure S2** Normalized MS1 TIC for log^­^_2_ transformed intensity values for LC-MS proteomics…..3

**Figure S3** Hierarchical clustering of onvansertib proteomics data showing that 72-hours with onvansertib exposure cluster entirely together………………………………………………………..4

**Figure S4** Volcano plots of (A) 1-hour, (B) 4-hour, and (C) 6-hour onvansertib exposure, showing only one differentially expressed protein amongst these entire time sets……………….5

**Figure S5** Venn Diagram of similar proteins between the 12-, 24-. 48-, and 72-hour samples….6

**Figure S6** Gene set enrichment analysis of the differentially expressed proteins after 12-hours of onvansertib exposure………………………………………………………………………………….7

**Figure S7** Gene set enrichment analysis of the differentially expressed proteins after 24-hours of onvansertib exposure………………………………………………………………………………….8

**Figure S8** Gene set enrichment analysis of the differentially expressed proteins after 48-hours of onvansertib exposure………………………………………………………………………………….9

**Figure S9** Gene set enrichment analysis of the differentially expressed proteins after 72-hours of onvansertib exposure………………………………………………………………………………..10

**Figure S10** Ingenuity pathway analysis of statistically significant proteins amongst the 12-, 24-, 48, and 72-hour time points…………………………………………………………………………….11

**Figure S11** Two different lipids that are not highly present in the early time points, but gradually increase over time with onvansertib exposure. (A) *m/z* 798.542 identified to be the [M+K^+^]^+^ of PC(34:1). (B) *m/z* 770.513 identified to be the [M+K^+^]^+^ of PC(32:1)………………………………12

**Figure S12** *m/z* 369.351, identified as the [M+H^+^-H_2_O]^+^ of cholesterol, as observed with MALDI-2 ionization……………………………………………………………………………………………….13

**Figure S13** MALDI MSMS of lipids found in the mass spectrometry images for validation of lipid identity. The above tandem MS spectra originate from the following precursors: (**A**) *m/z* 796.464, (**B**) *m/z* 798.542, and (**C**) *m/z* 770.513………………………………………………………………..14

**Figure S14** MALDI-2 MSMS of lipids found in the mass spectrometry images for validation of lipid identity. The above tandem MS spectra originate from the precursor *m/z* 808.585………15

**Figure S15** Segmentation analysis of spheroids from CHCA and DHB experiments used to ascertain average peak areas for masses of interest ……………………………………..………16


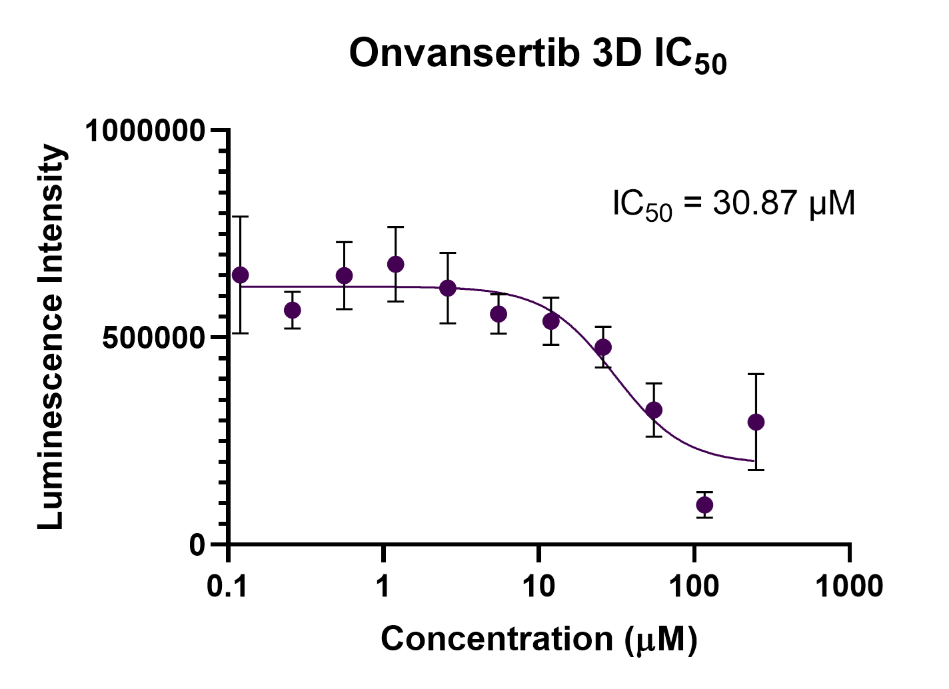


**Figure S1** IC_50_ curve of HCT 116 spheroids incubated with onvansertib for 72 hours.

| **Parameters** | **DHB** | **CHCA** |
| --- | --- | --- |
| Nozzle Temperature (°C) | 75 | 70 |
| Passes | 8 | 14 |
| Flow Rate (mL/min) | 0.10 | 0.12 |
| Velocity (mm/min) | 1,000 | 800 |
| Track Spacing (mm) | 2 | 2 |
| Pressure (psi) | 10 | 10 |
| Gas Flow Rate (L/min) | 3 | 3 |
| Nozzle Height (mm) | 40 | 40 |
| Drying Time (sec) | 20 | 10 |

**Table S1** Spray parameters for MALDI MSI experiments with either DHB or CHCA.


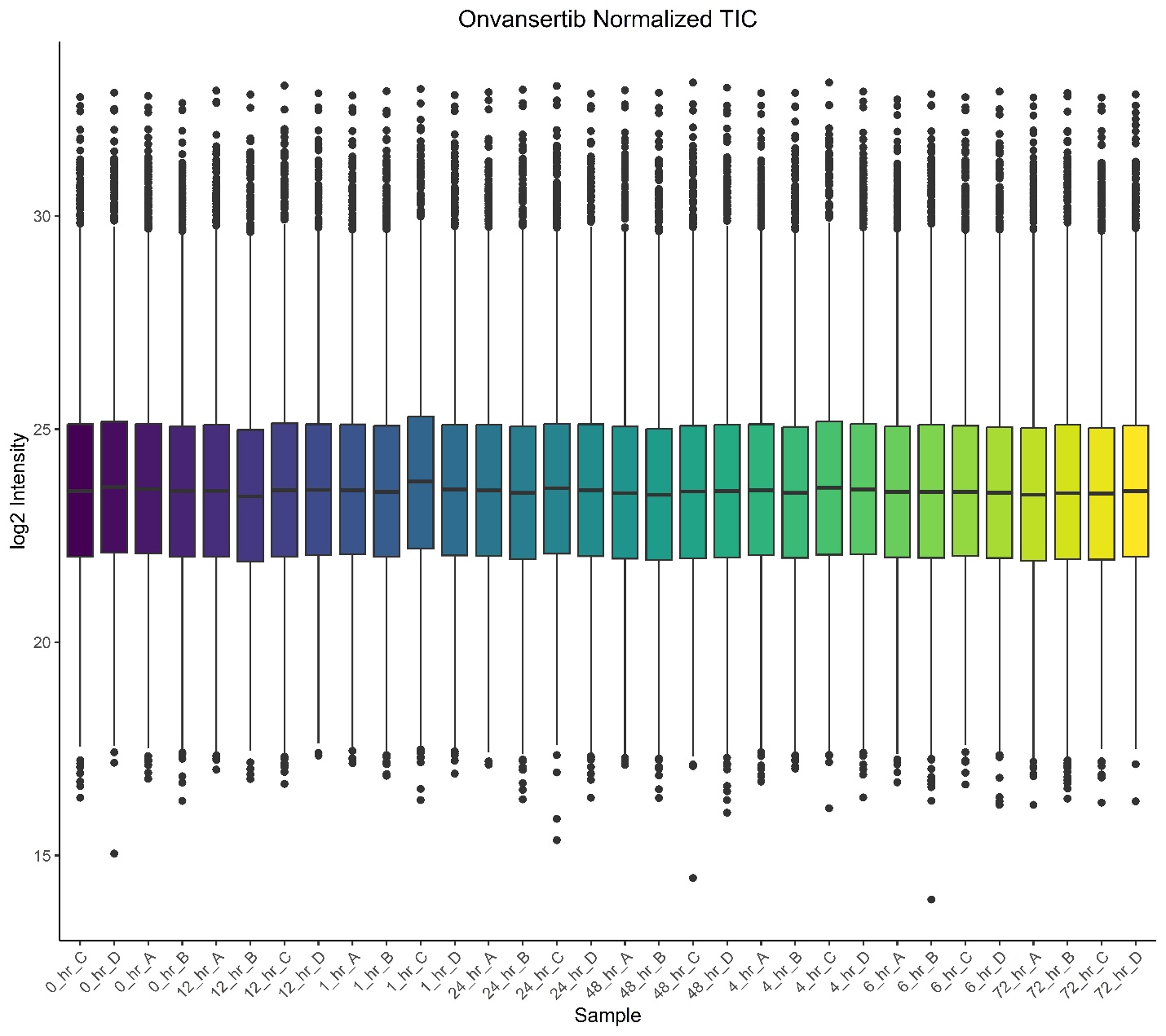


**Figure S2** Normalized MS1 TIC for log^­^_2_ transformed intensity values for LC-MS proteomics.


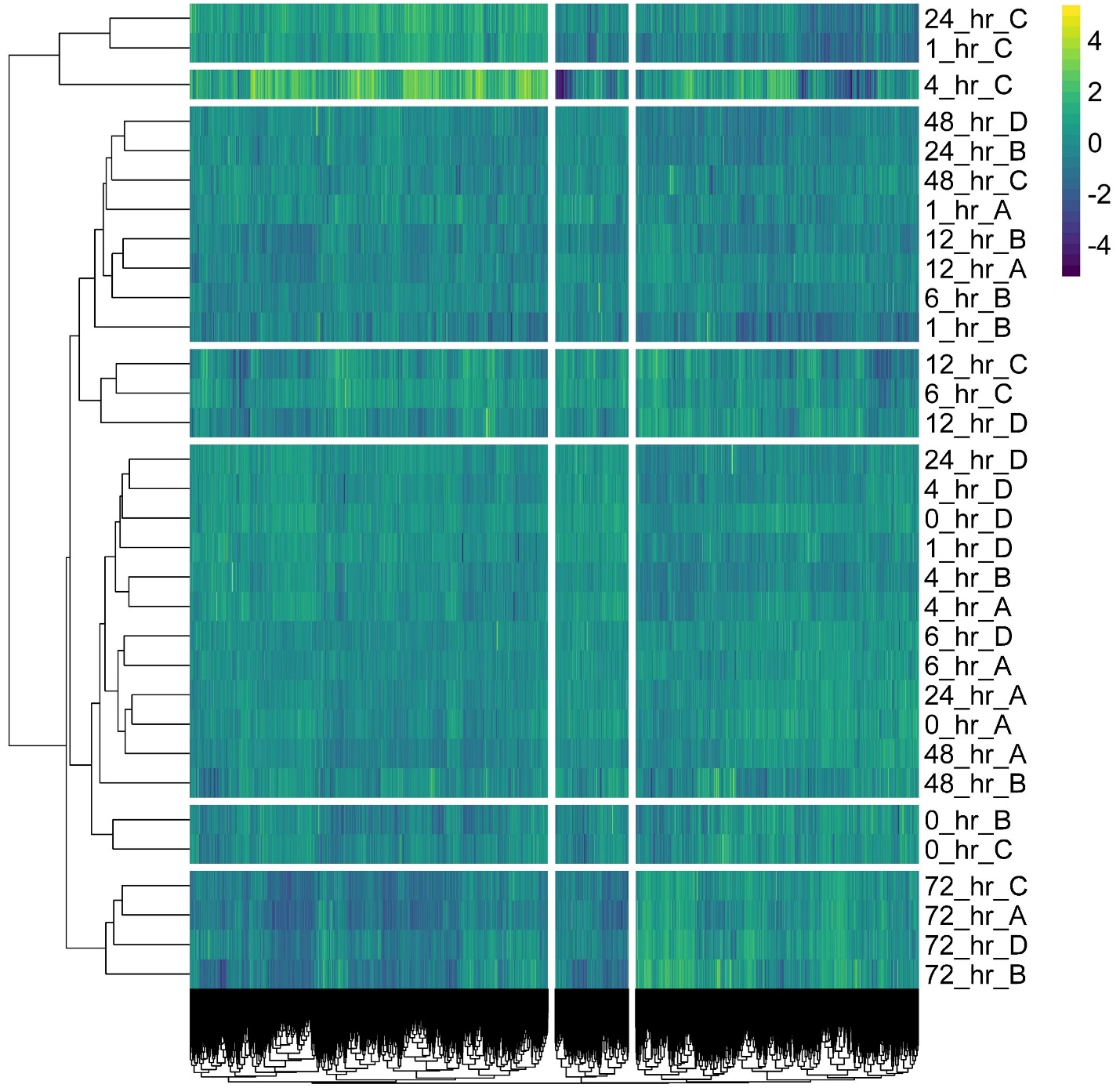


**Figure S3** Hierarchical clustering of onvansertib proteomics data showing that 72-hours with onvansertib exposure cluster entirely together.


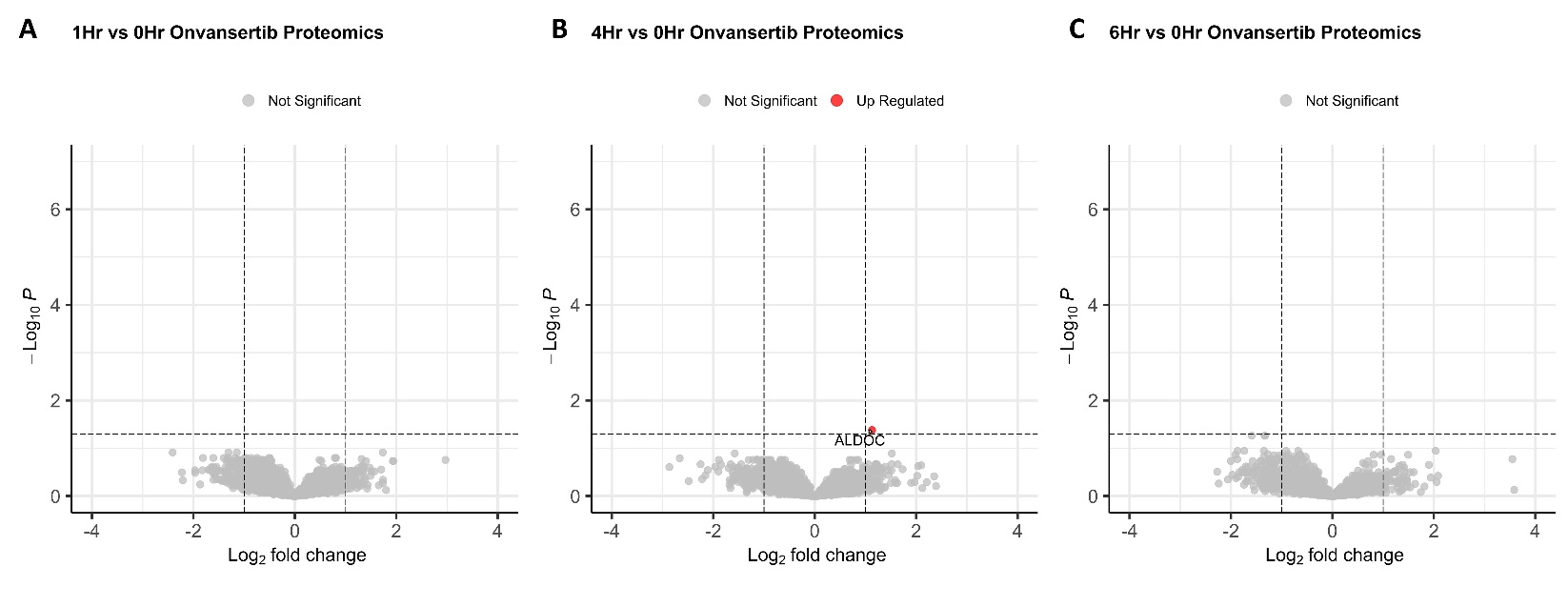


**Figure S4** Volcano plots of (A) 1-hour, (B) 4-hour, and (C) 6-hour onvansertib exposure, showing only one differentially expressed protein amongst these entire time sets.


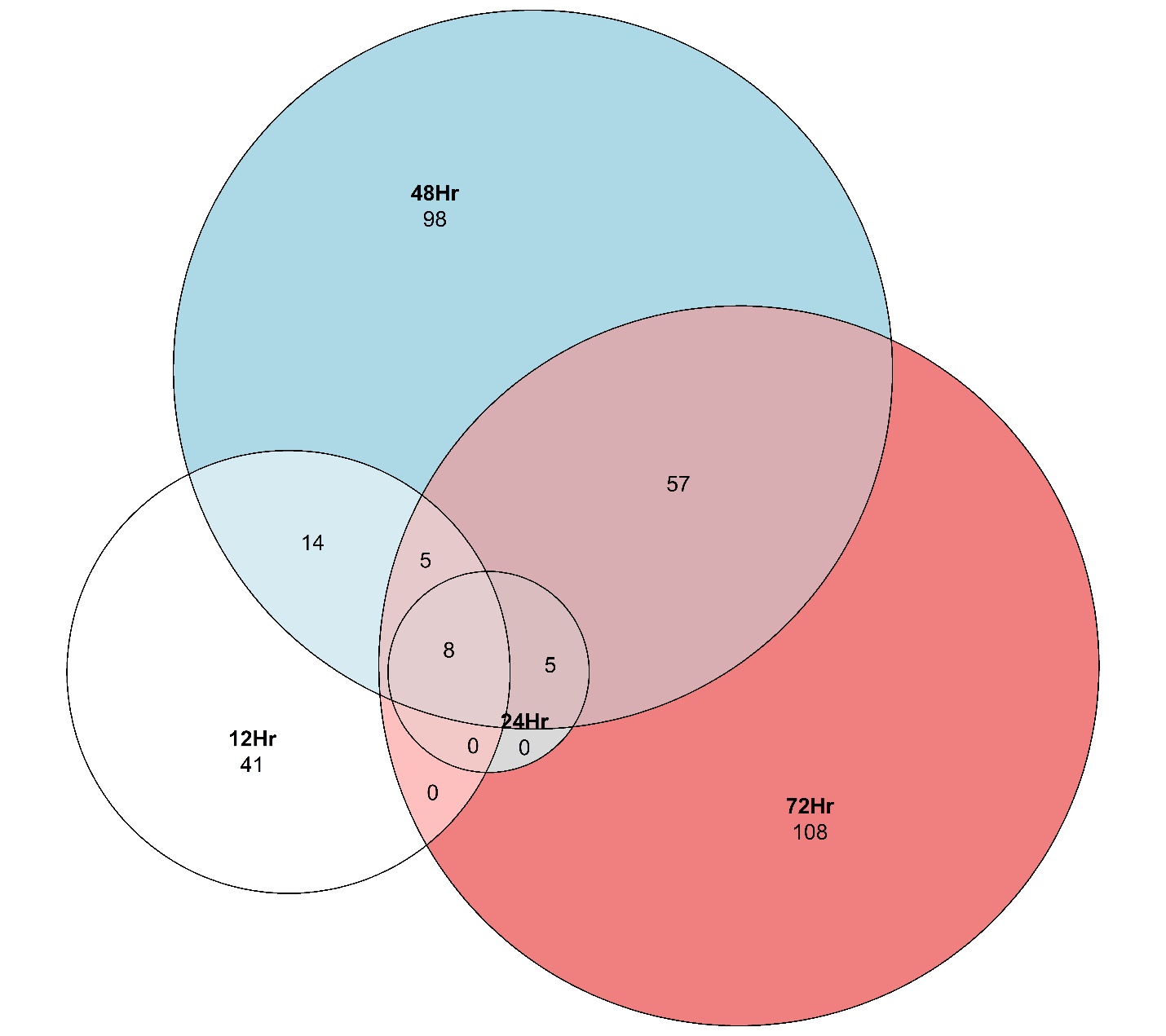


**Figure S5** Venn Diagram of similar proteins between the 12-, 24-. 48-, and 72-hour samples.


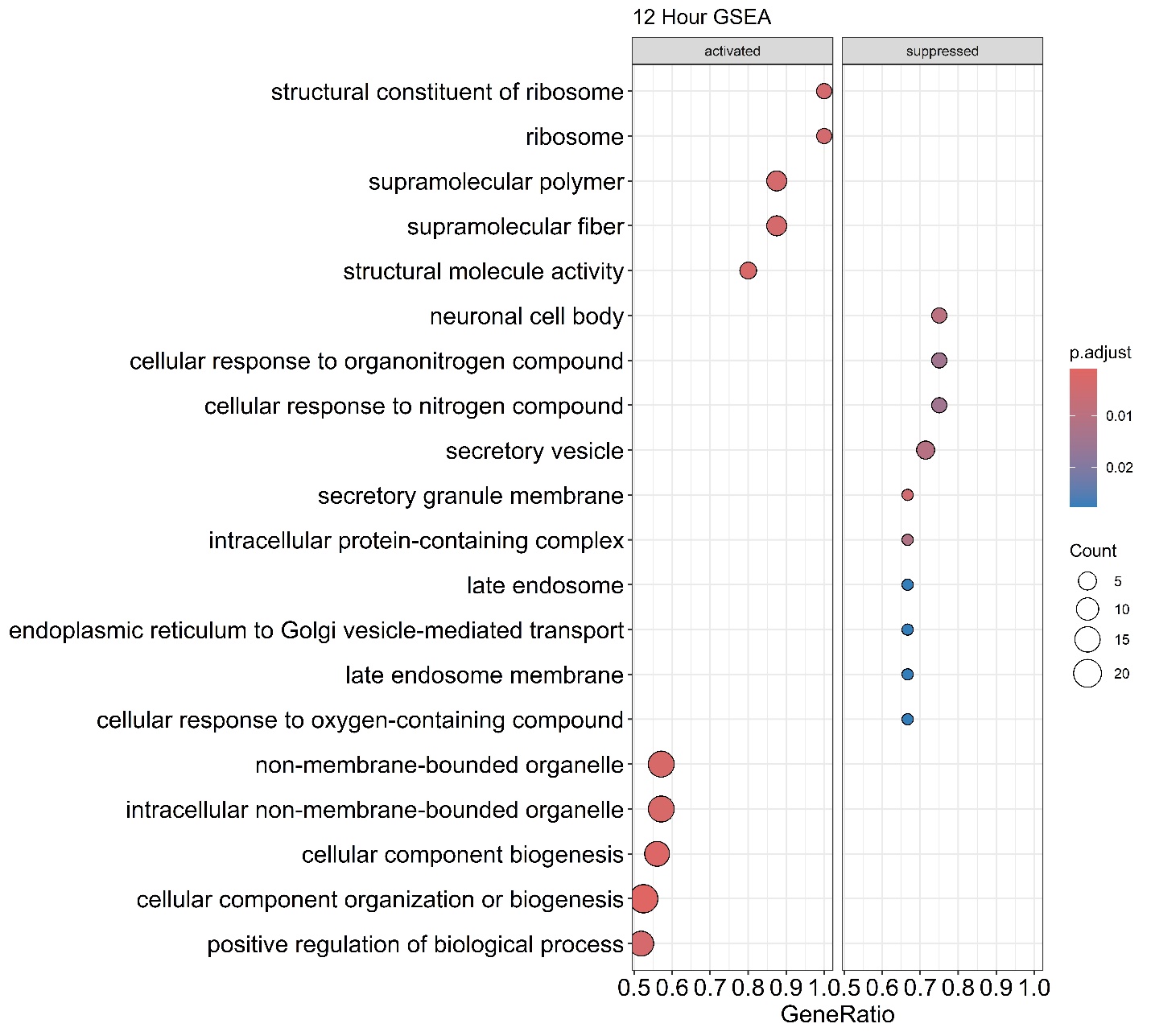


**Figure S6** Gene set enrichment analysis of the differentially expressed proteins after 12-hours of onvansertib exposure.


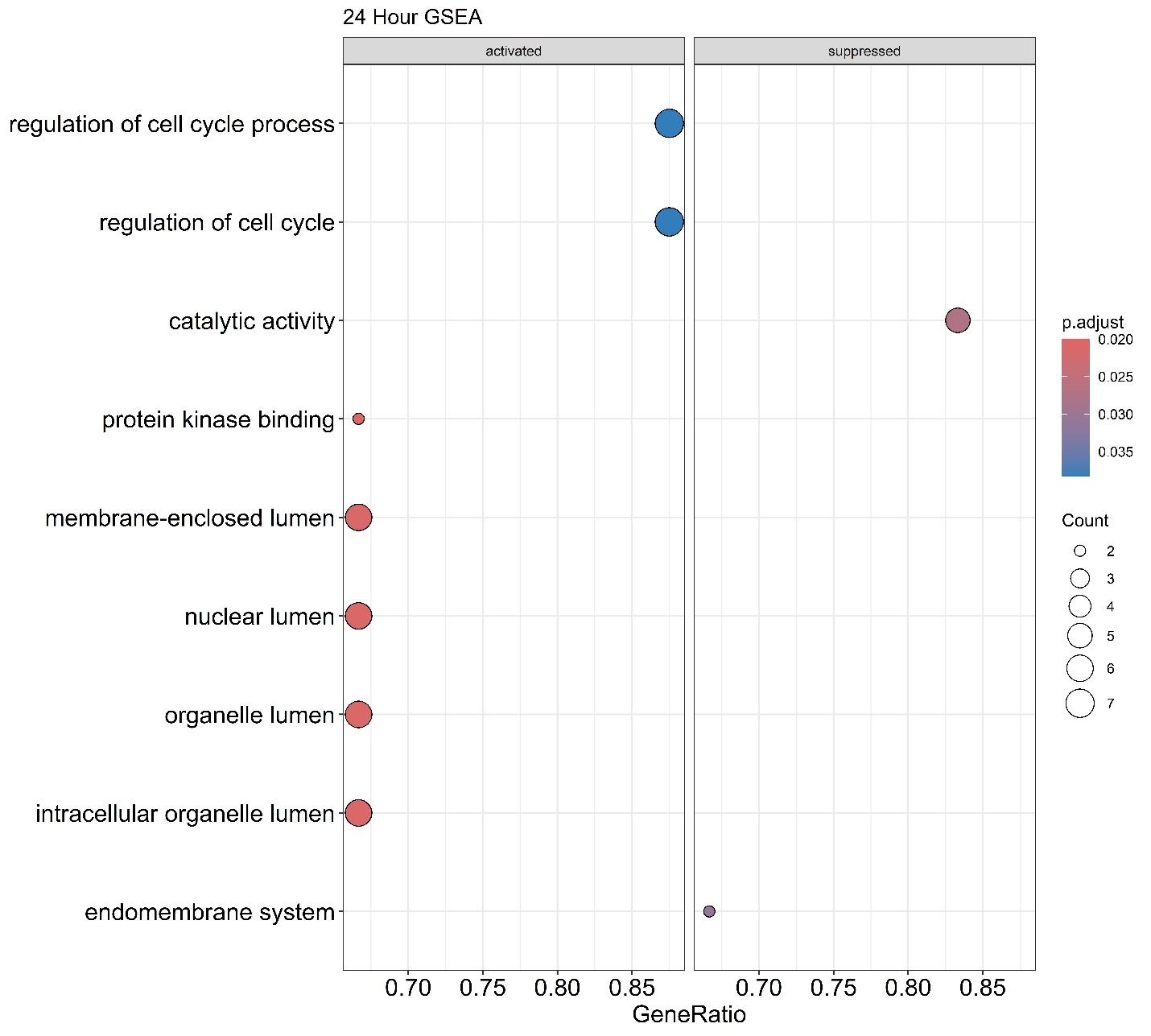


**Figure S7** Gene set enrichment analysis of the differentially expressed proteins after 24-hours of onvansertib exposure.


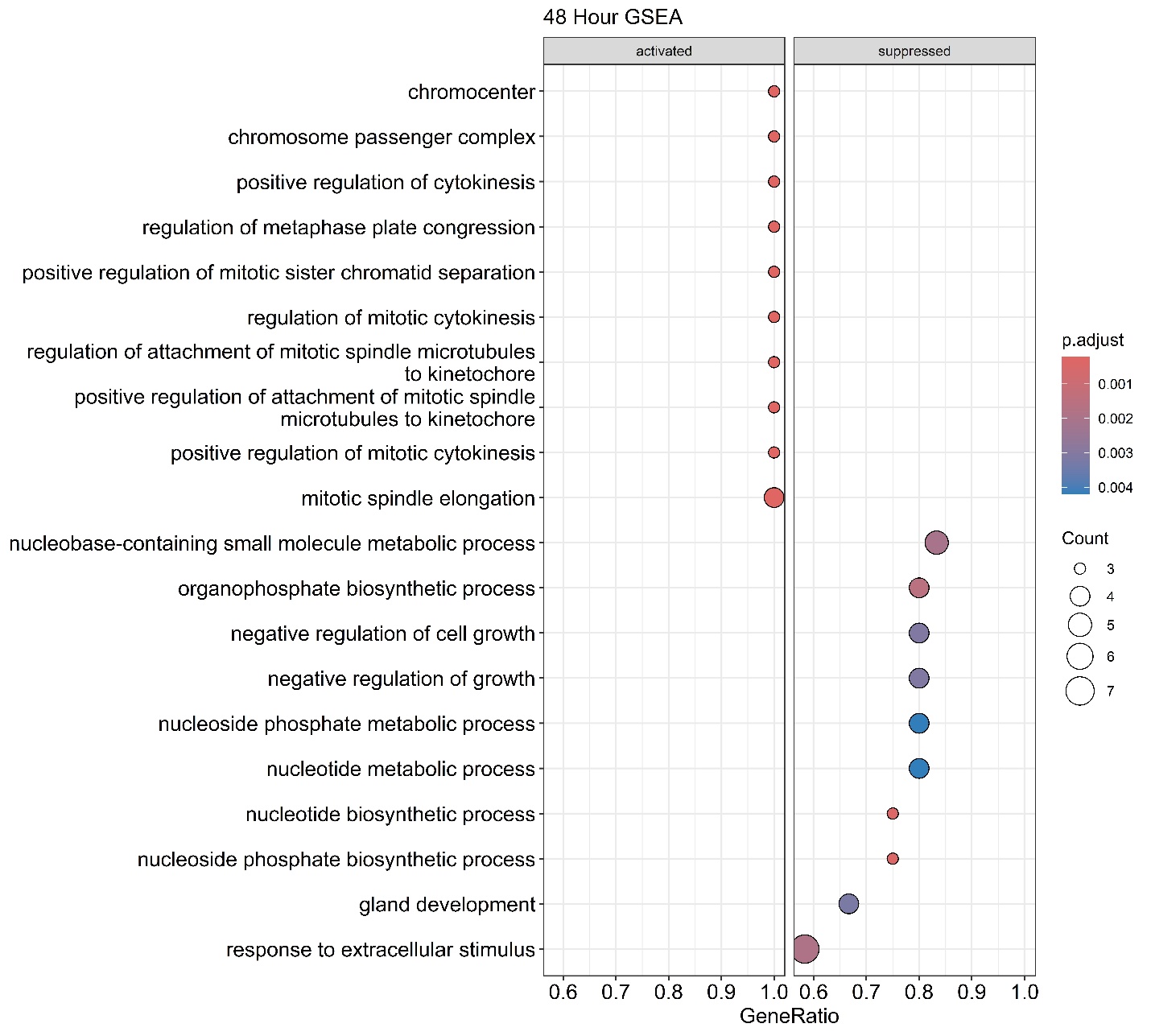


**Figure S8** Gene set enrichment analysis of the differentially expressed proteins after 48-hours of onvansertib exposure.


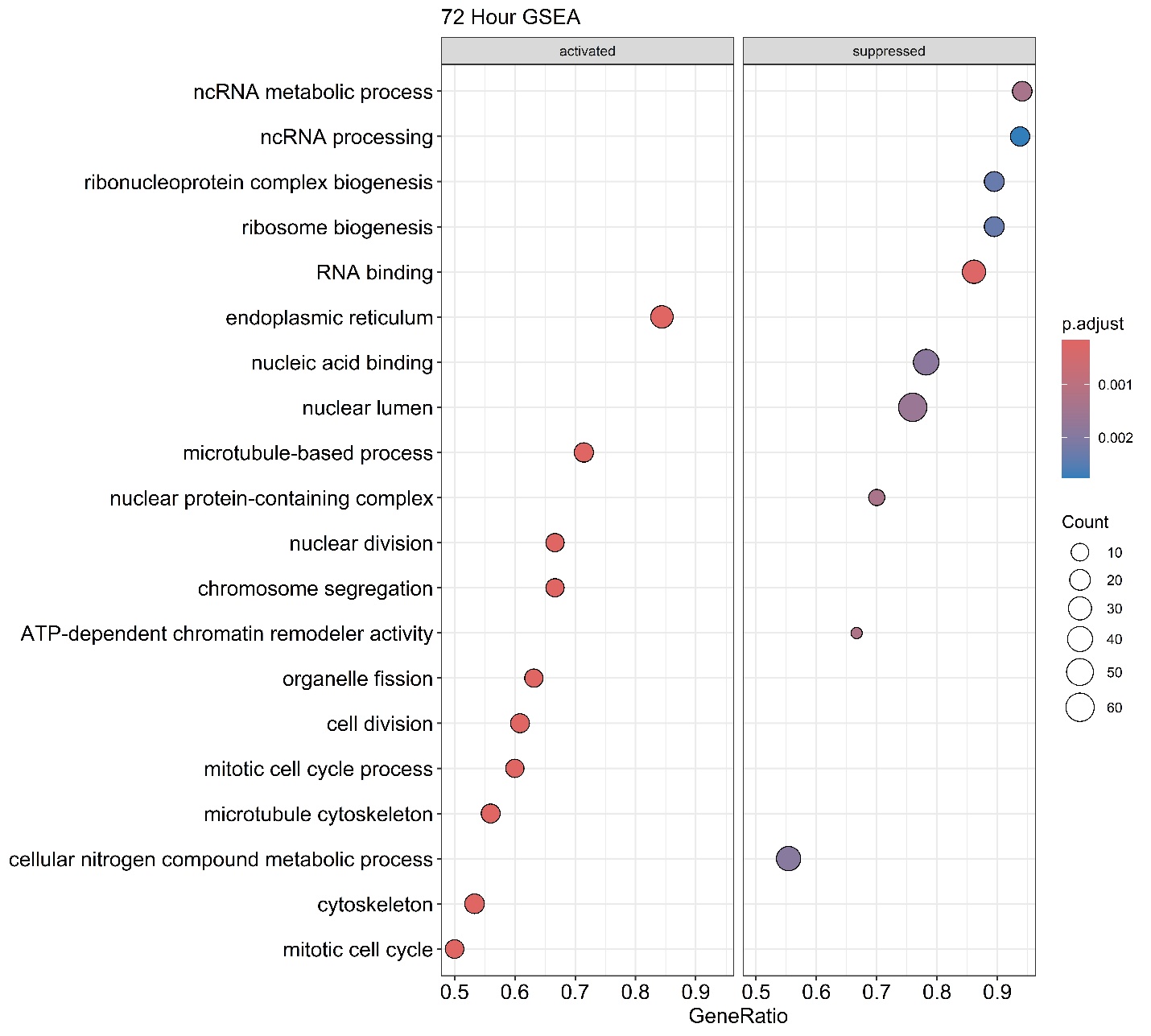


**Figure S9** Gene set enrichment analysis of the differentially expressed proteins after 72-hours of onvansertib exposure.


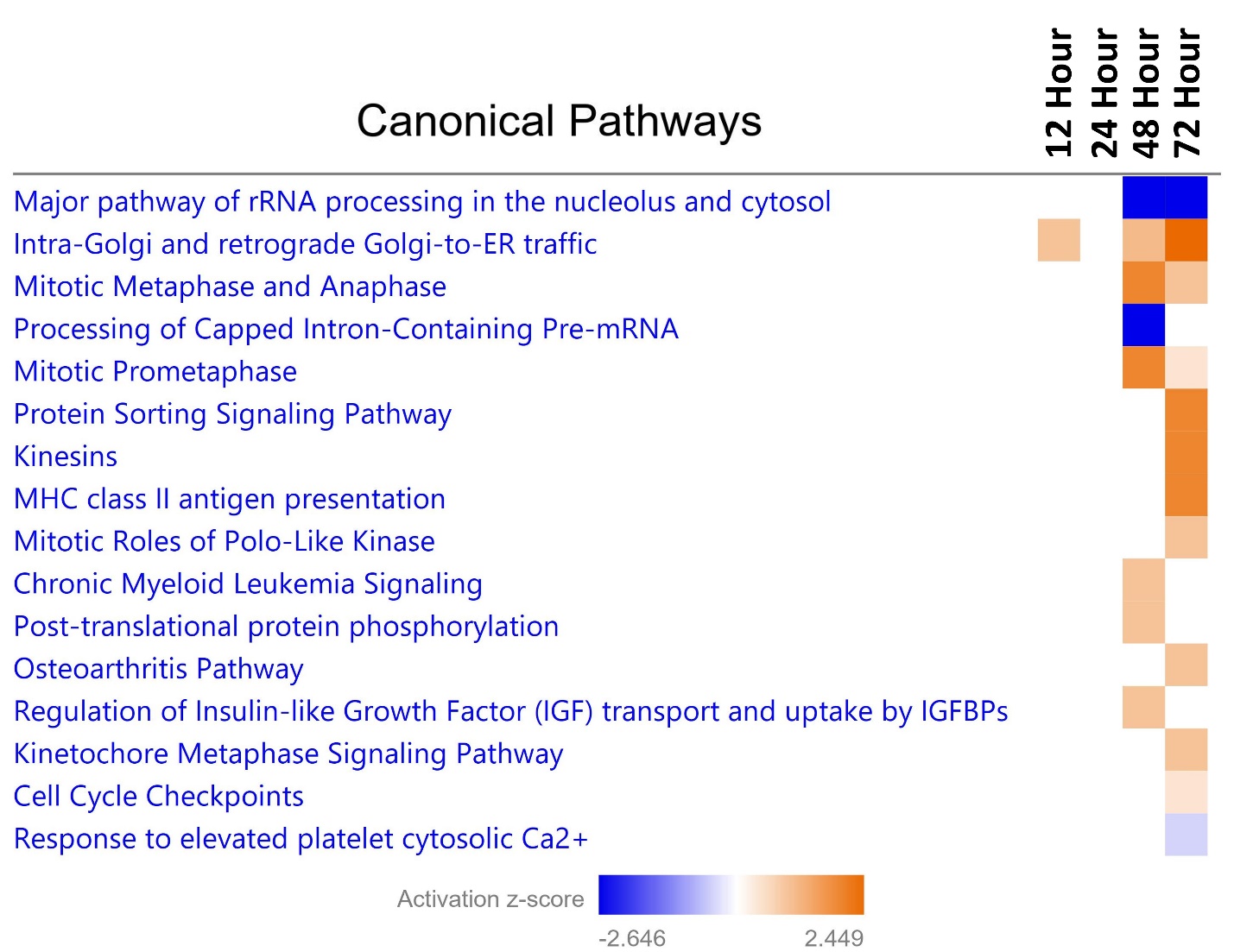


**Figure S10** Ingenuity pathway analysis of statistically significant proteins amongst the 12-, 24-, 48, and 72-hour time points.


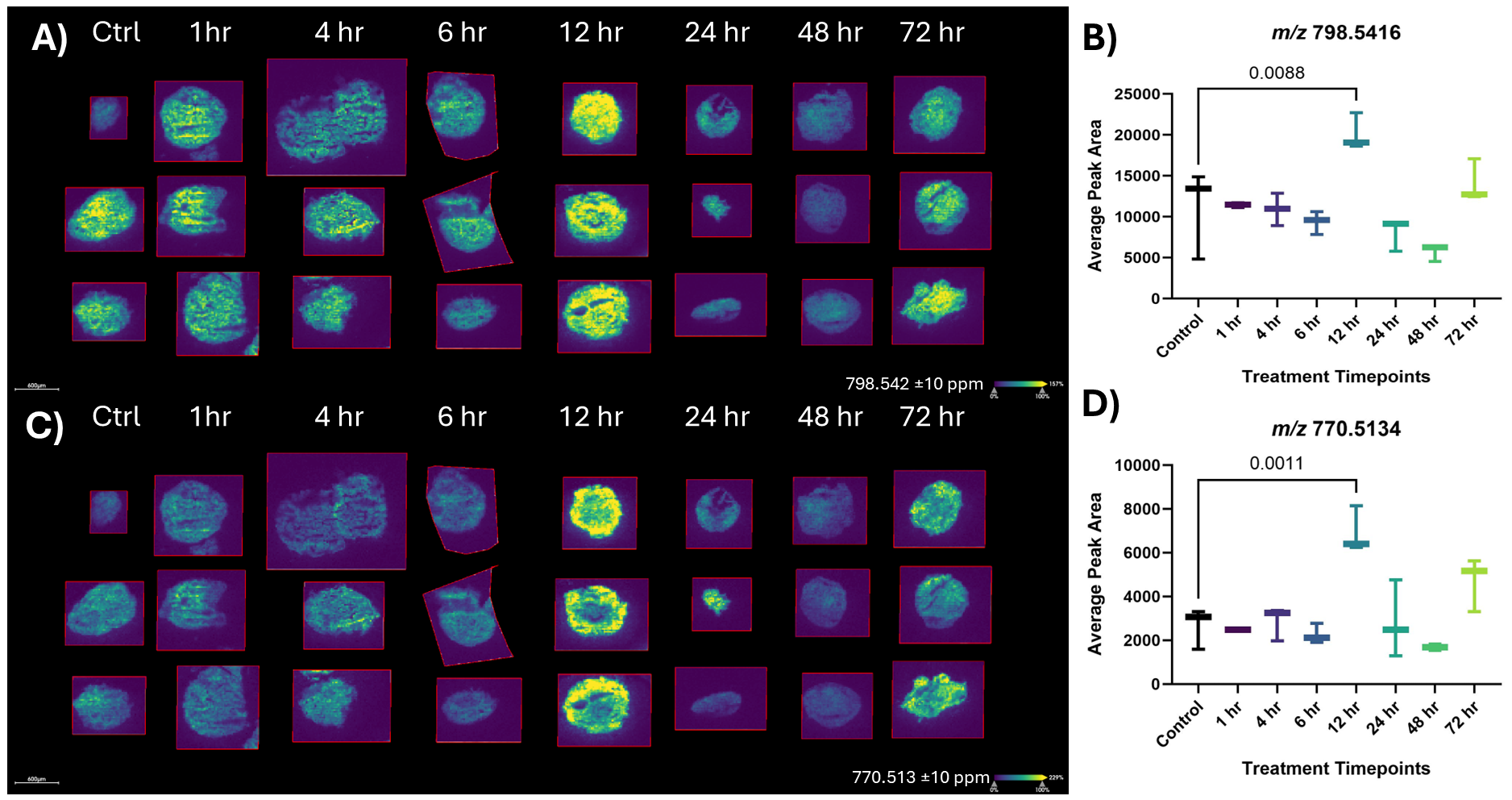


**Figure S11** Two different lipids that are not highly present in the early time points, but gradually increase over time with onvansertib exposure. (**A**) *m/z* 798.542 Da identified to be the [M+K]^+^ of PC(34:1). (**B**) Average peak area observed at *m/z* 798.542 Da for the segmented spheroid data with ANOVA analyses displayed against controls. (**C**) *m/z* 770.513 Da identified to be the [M+K]^+^ of PC(32:1). (**D**) Average peak area observed at *m/z* 770.513 Da for the segmented spheroid data with ANOVA analyses displayed against controls.


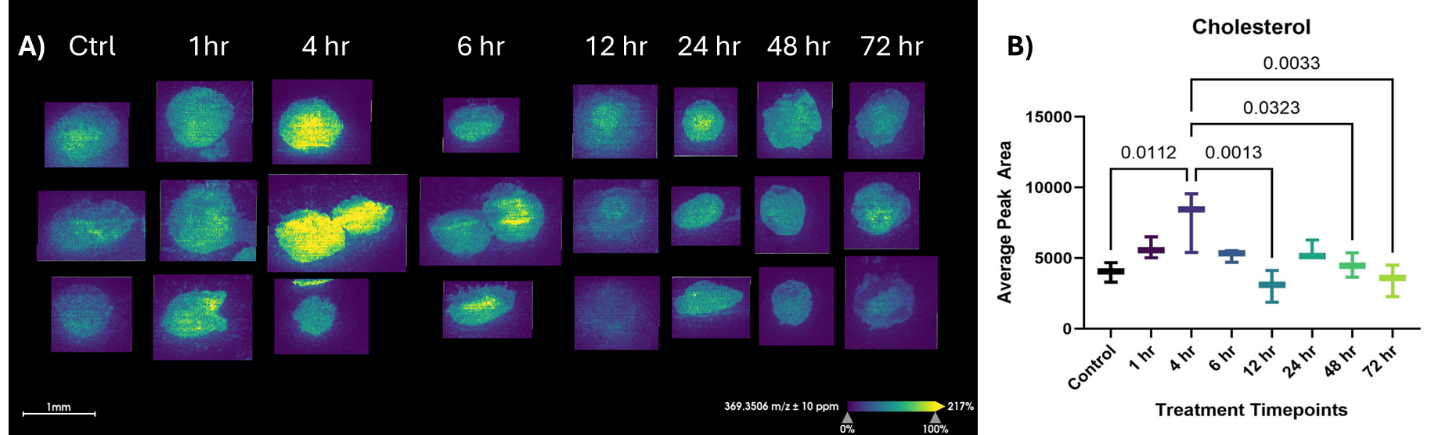


**Figure S12 (A)** *m/z* 369.351 Da, identified as the [M+H-H_2_O]^+^ of cholesterol, as observed with MALDI-2 ionization. (**B**) Plot of average peak areas found for spheroids with ANOVA multi-comparison analyses.


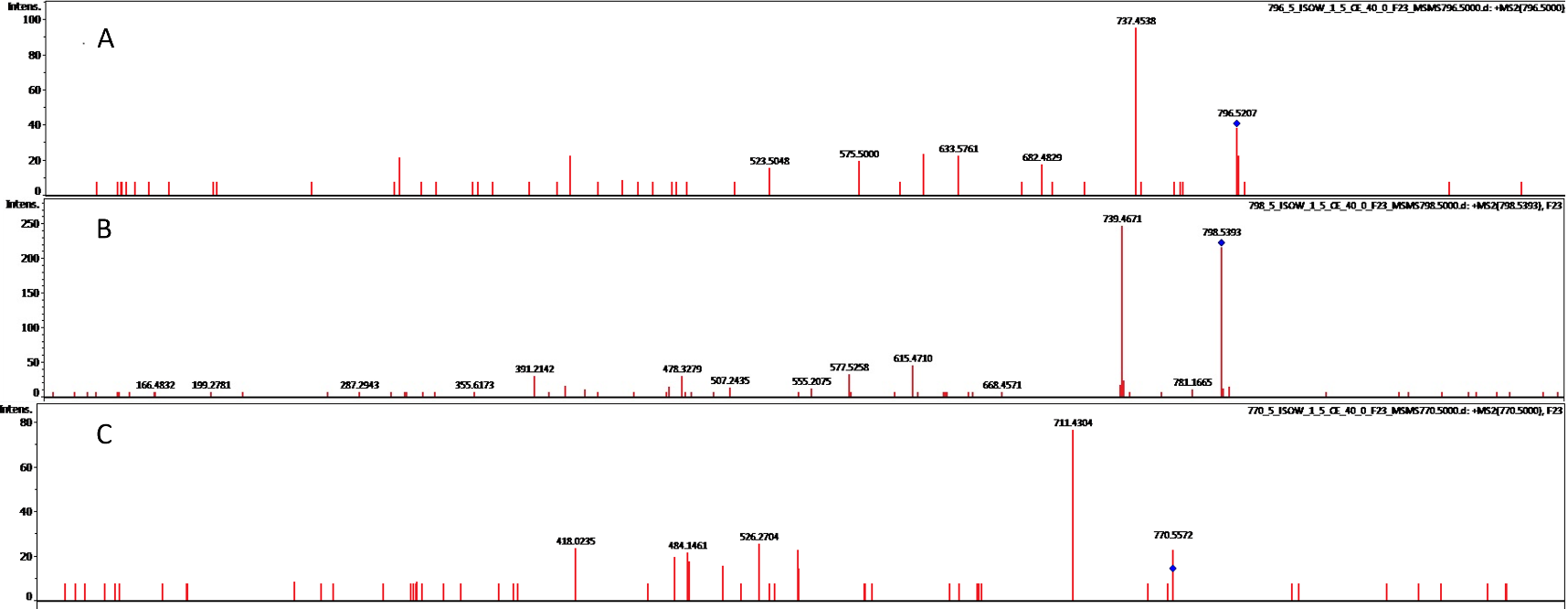


**Figure S13** MALDI MSMS of lipids found in the mass spectrometry images for validation of lipid identity. The above tandem MS spectra originate from the following precursors: (**A**) *m/z* 796.464 fragmented at 40eV, (**B**) *m/z* 798.542 fragmented at 40Ev, and (**C**) *m/z* 770.513 fragmented at 40 eV.

59.0669


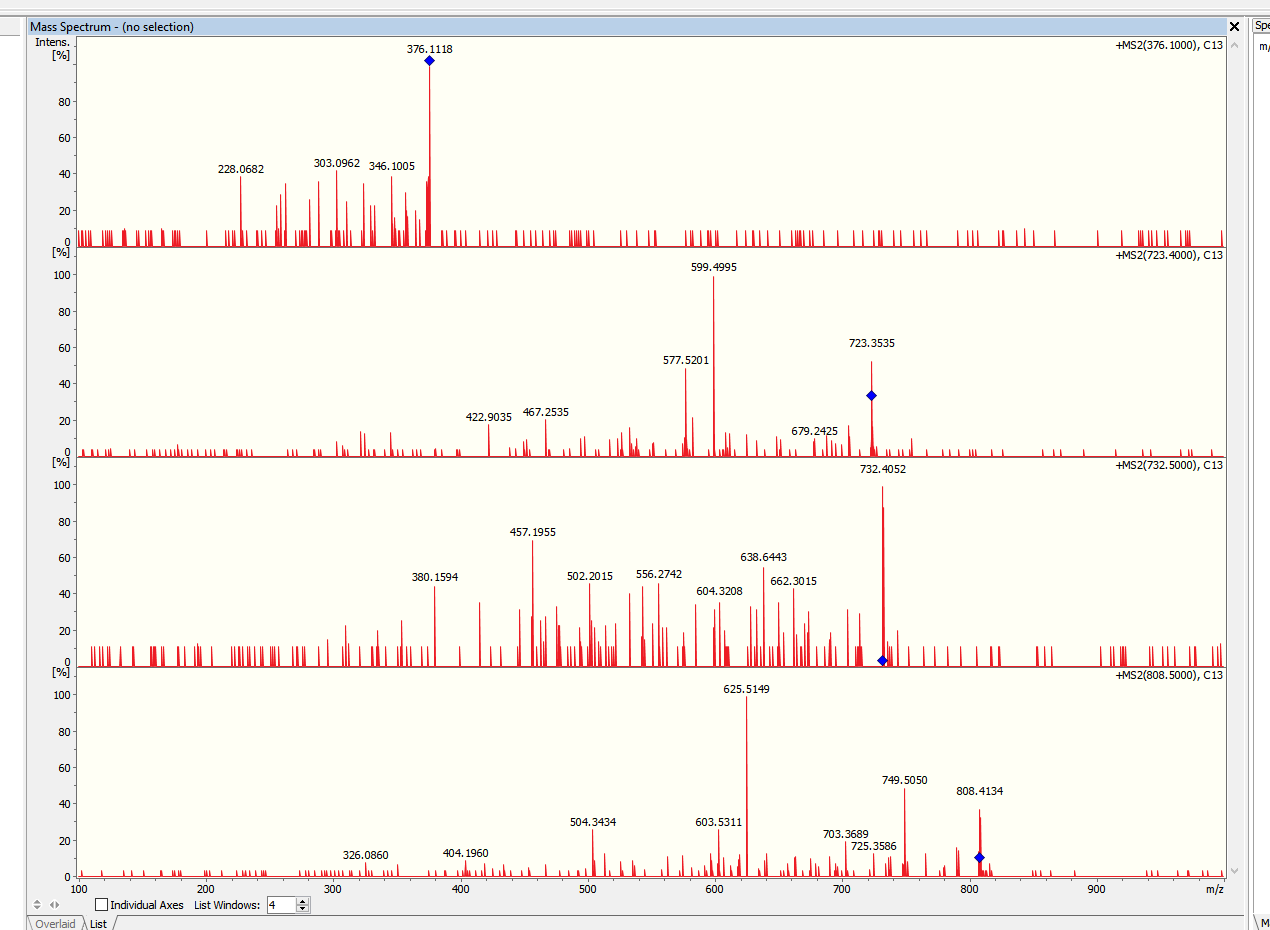


**Figure S14** MALDI-2 MSMS of lipids found in the mass spectrometry images for validation of lipid identity. The above tandem MS spectra originate from the precursor *m/z* 808.585 fragmented at 40 eV.


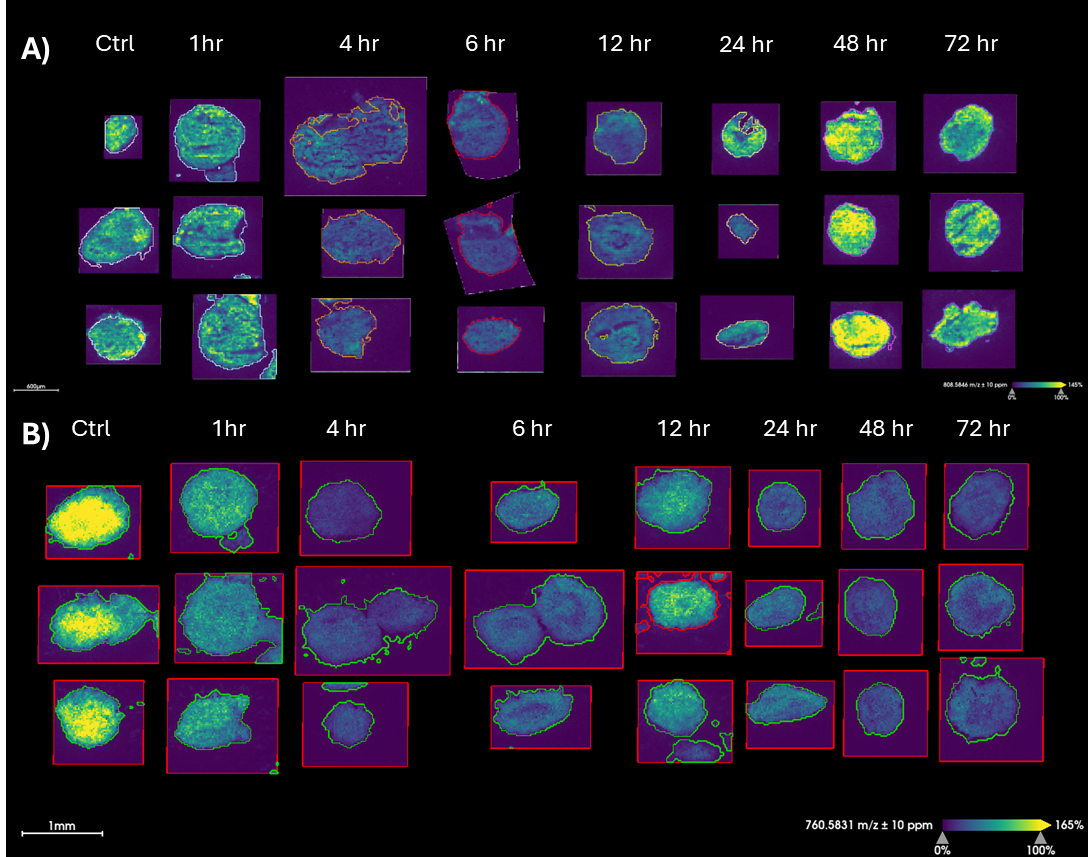


**Figure S15** Data segmentation completed to determine average peak area signal from spheroids. (A) Spheroids analyzed with CHCA matrix. (B) Spheroids analyzed with DHB matrix.
